# β-Barrel Nanopores with an Acidic–Aromatic Sensing Region Identify Proteinogenic Peptides at Low pH

**DOI:** 10.1021/acsnano.1c11455

**Published:** 2022-03-18

**Authors:** Roderick Corstiaan Abraham Versloot, Sabine Angenieta Paulina Straathof, Gemma Stouwie, Matthijs Jonathan Tadema, Giovanni Maglia

**Affiliations:** Groningen Biomolecular Sciences and Biotechnology Institute, University of Groningen, Groningen, Groningen 9747AG, Netherlands

**Keywords:** nanopores, protein sequencing, nanopore spectrometry, nanopore-forming toxins, single-molecule

## Abstract

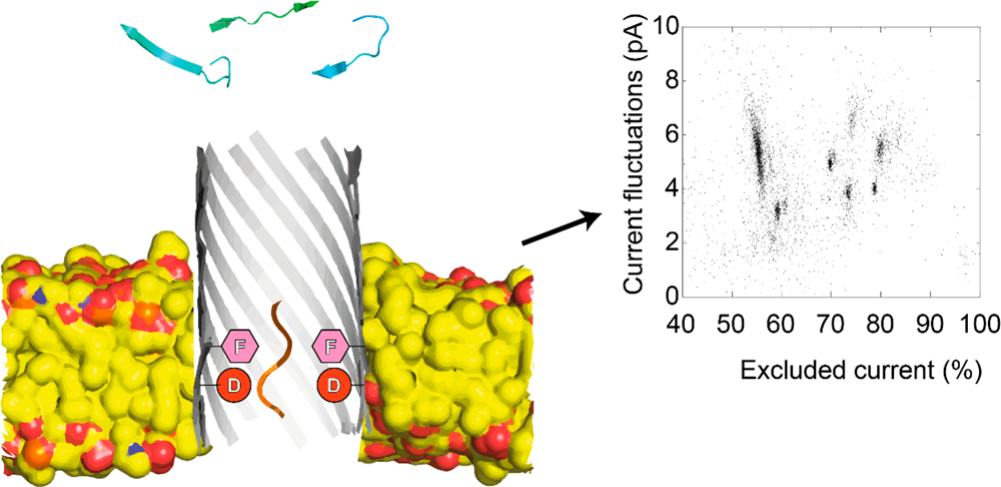

Biological nanopores are emerging as sensitive single-molecule sensors for proteins and peptides. The heterogeneous charge of a polypeptide chain, however, can complicate or prevent the capture and translocation of peptides and unfolded proteins across nanopores. Here, we show that two β-barrel nanopores, aerolysin and cytotoxin K, cannot efficiently detect proteinogenic peptides from a trypsinated protein under a wide range of conditions. However, the introduction of an acidic–aromatic sensing region in the β-barrel dramatically increased the dwell time and the discrimination of peptides in the nanopore at acidic pH. Surprisingly, despite the fact that the two β-barrel nanopores have a similar diameter and an acidic–aromatic construction, their capture mechanisms differ. The electro-osmotic flow played a dominant role for aerolysin, while the electrophoretic force dominated for cytotoxin K. Nonetheless, both β-barrel nanopores allowed the detection of mixtures of trypsinated peptides, with aerolysin nanopores showing a better resolution for larger peptides and cytotoxin K showing a better resolution for shorter peptides. Therefore, this work provides a generic strategy for modifying nanopores for peptide detection that will be most likely be applicable to other nanopore-forming toxins.

The development of diagnostics devices for personalized healthcare relies on the use of low-cost and portable devices that are capable of addressing biological molecules.^1^ In proteomics, tandem mass spectrometry (MS) is the gold standard for the detection and sequencing of proteins due to its precision and ability to measure complex samples.^2^ A MS, however, is a large and expensive machine that most often requires specialized facilities to operate.^3,4^ Furthermore, MS requires very a low pressure (vacuum) to operate, which at present cannot be easily integrated into portable devices.

By contrast, biological nanopores have great potential for making low-power and low-cost devices as they are capable of high-throughput measurements^5^ and can address single molecules even in complex solutions.^6−9^ In a typical nanopore measurement, two fluidic compartments filled with an electrolyte solution are separated by a nonconducting membrane containing a single nanopore. The ionic current through the nanopore is measured when a potential difference is applied over the membrane. When analytes enter the nanopore, the open nanopore current (*I*_O_) is transiently reduced, and the current blockade provides information on the size of the analyte present in the nanopore.

Biological nanopores have been used to capture folded proteins,^10−12^ and the nanopore signal has been associated with the size, mass, volume, and shape of the protein.^13−16^ However, the capture and translocation of proteins across the nanopore is complicated by the complex three-dimensional structure and nonuniform charge distribution of the protein.^17^ Recently, we have shown that coupling the nanopore to an unfoldase complex could provide a generic method for controlling the feed of proteins to the nanopore.^18^ Single proteins are then addressed by directly reading the unfolded protein as it translocated across the nanopore or by chopping the protein into peptides and reading the fragments in sequence.^19^ We also showed that the latter “chop and drop” method could potentially discriminate among the majority of proteins of the human proteome, provided that most peptides were accurately detected by the nanopore.^20^ α-Helical fragaceatoxin C (FraC) nanopores were shown to efficiently capture peptides in low pH conditions regardless of their charge.^19,21−23^ In turn, this allowed several proteins to be recognized by measuring the peptide spectrum originating from trypsinated proteins. Although this approach is capable of distinguishing among proteins at the single-molecule level, it is unclear whether a cylindrical β-barrel nanopore, such as those incorporated into the nanopore–unfoldase complex, can be engineered to identify proteinogenic peptides in a fashion similar to conical α-helical FraC nanopores.

Previous work on peptide detection in β-barrel nanopores suggested that peptides might be sampled. Early work focused on the interaction of designed structured peptides with an α-hemolysin (αHL) nanopore.^24,25^ In another example, a mutant of α-hemolysin (M133F) was engineered to detect aromatic peptides.^26^ More recently, wild-type αHL was used to discriminate between small positively charged peptides and showed a correlation between the blockade depth and the mass of the peptide.^27^ Aerolysin has emerged as a favorite β-barrel nanopore for peptide analysis. The nanopore was shown to detect differences in length^28^ and composition^29^ in uniformly charged peptides. In one example, the differences among almost all 20 amino acids in polyarginine peptides were observed.^30^ Aerolysin mutants were shown to detect a range of model negatively charged peptides^31^ as well as phosphorylation and acetylation on τ-peptides.^32,33^ In a recent communication, the aerolysin nanopore was engineered to detect negative and weakly positive peptides at near-physiological pH values.^34^

Here, we assessed the ability of two β-barrel nanopores, aerolysin and cytotoxin K (CytK), to capture a mixture of peptides originating from the protease digestion of lysozyme. We showed that in 1 M KCl solutions the wild-type nanopores could not resolve peptide mixtures at physiological or acidic pH values. The introduction of an acidic–aromatic sensing region in the β-barrel, however, allows the entry and greatly enhances the dwell time of the peptides inside the nanopore. The acidic–aromatic aerolysin nanopore could differentiate the six peptides originating from lysozyme digestion with a mass range of 1000–1700 Da without any overlap between the peptide clusters in the nanopore spectrum. We found that the peptide capture of aerolysin at low pH values is governed by the electro-osmotic flow, whereas the peptide capture in CytK is based on electrophoresis. Despite the difference in their capture mechanisms, a similar set of mutations was required to improve peptide detection, showing that our strategy might also be used to adept other β-barrel nanopores for peptide detection. Extremely sensitive β-barrel nanopore peptide sensors might ultimately be integrated with unfoldase and peptidase complexes to allow the single-molecule measurement of proteins.^18,20^

## Results and Discussion

### Aerolysin Engineering for Nanopore Peptide Spectroscopy

**Figure 1:**
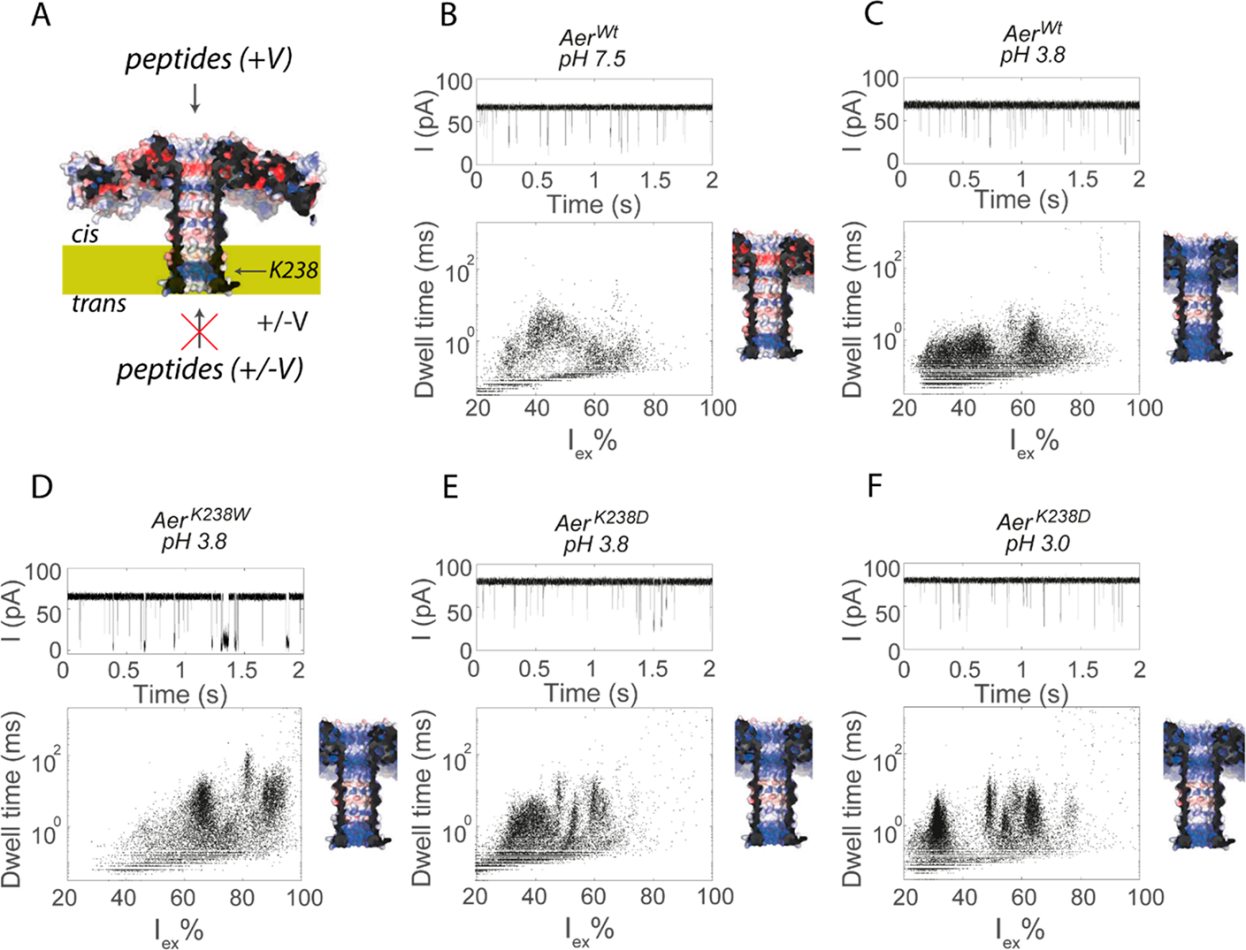
Detection of trypsinated lysozyme by aerolysin. (A) Cut-through structure of the aerolysin nanopore (PDB 5JZT) shown as surface potential maps with positively or negatively charged regions in blue and red, respectively. (B–F) Ionic current traces (top) and dwell time vs excluded current (bottom left) for aerolysin nanopores under different pH values. The figures on the right indicate the potential map of the charge distribution inside the nanopore at different pH values. Peptides were tested by adding 4 μg of trypsinated lysozyme to the *cis* compartment (400 μL). The final concentration of trypsinated lysozyme was 10 ng/μL. Data were recorded in 1 M KCl at an applied potential of +150 mV at the indicated pH.

We tested the ability of the wild-type aerolysin (Aer^Wt^, Figure 1A and Figure S1) nanopore to characterize peptides by adding a mixture of peptides that resulted from the trypsination of lysozyme from *Gallus gallus* to either side of the nanopore in 1 M KCl at either pH 7.5 (Figure 1B) or pH 3.8 (Figure 1C). When added to the *cis* compartment, the mixture induced fast current blockades as a result of the entry of some of the peptides into the nanopore under a positive applied potential. The addition of the peptide mixture to the *trans* side did not induce peptide blockades at either the positive applied potential or the negative applied potential. Although Aer^Wt^ captured peptides more efficiently at pH 3.8, the peptides were badly resolved, as the clusters were broad and showed significant overlap. To improve the peptide recognition at low pH values, we reduced the positive charge in the nanopore by substituting Lys238 on the *trans* side of the β-barrel of aerolysin with four different residues: Aer^K238Q^ and Aer^K238D^ to alter the charge and Aer^K238F^ and Aer^K238W^ to introduce aromatic residues. We tested the mutants for their ability to capture trypsinated lysozyme at pH 3.8 (Figures 1D and E and S2). All mutations increased the average dwell time of the lysozyme peptides in the nanopore, but Aer^K238W^ increased the dwell time the most (Figure S2C). Furthermore, Aer^K238W^ also shifted the excluded current (*I*_ex_% = (*I*_O_ – *I*_B_/*I*_O_) × 100%, where *I*_B_ is the ionic current during the event and *I*_O_ the is the current of the open nanopore) of the peptides to higher values, while the open nanopore current changed only slightly (64.7 ± 1.2 pA for Aer^K238W^ compared to 67.8 ± 1.0 pA for Aer^Wt^, +150 mV). Current versus voltage (*IV*) curves showed only slightly altered conductances for the different mutants compared to that of the wild type nanopores (Figure S3). Despite the longer dwell times of the peptides inside the nanopore, the resolution of the nanopores remained too poor to discriminate the mixture of lysozyme peptides.

**Figure 2:**
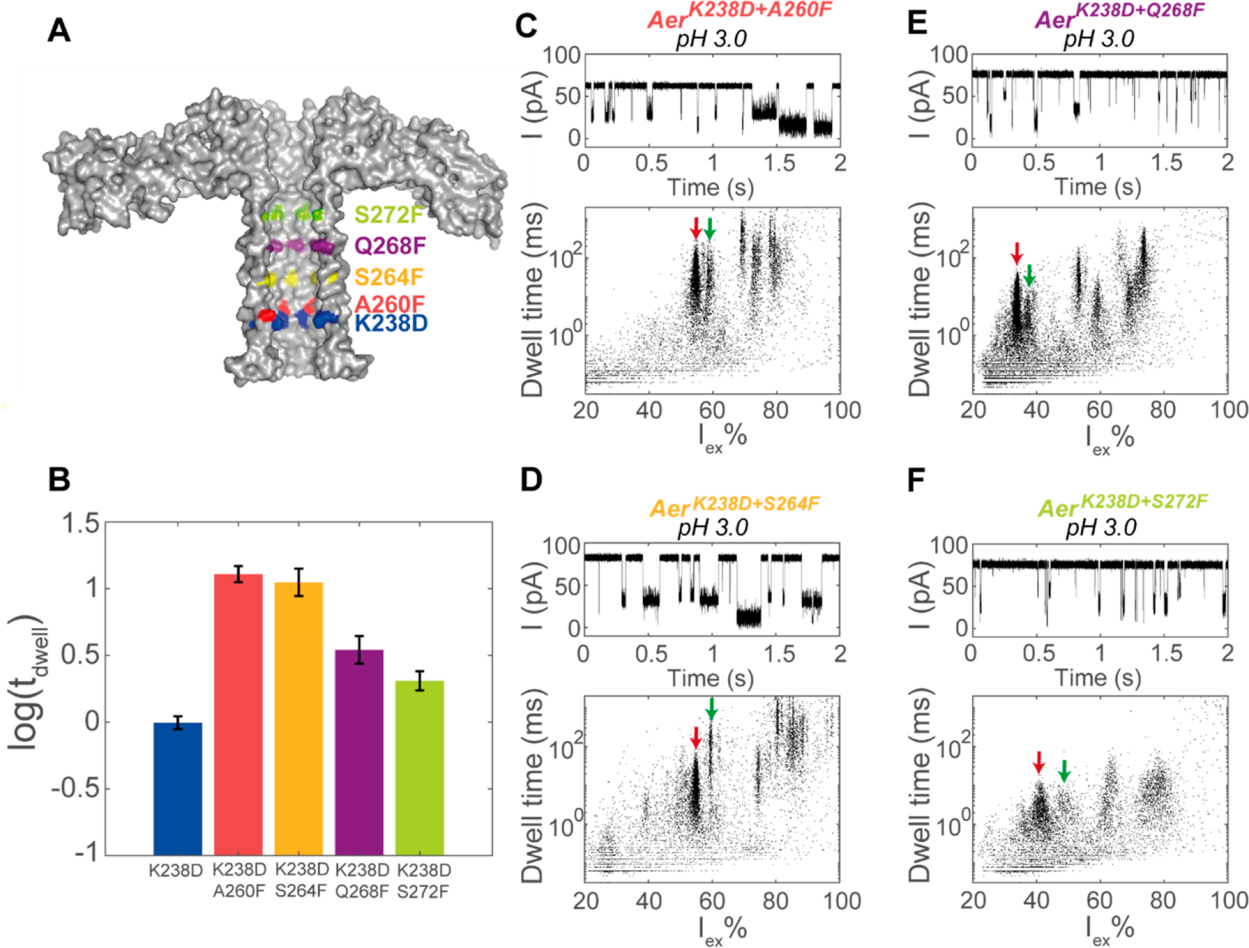
Detection of trypsinated lysozyme in the phenylalanine mutants at pH 3.0. (A) Cut-through of aerolysin (PDB 5JZT) showing the locations of mutated residues in the β-barrel. (B) Average log_10_(dwell time) of the events from trypsinated lysozyme in aerolysin mutants. The error bars indicate the standard error of the log_10_(dwell time) between three measurements in different nanopores. (C–F) Ionic current trace and event characteristics after the addition of 4 μg of trypsinated lysozyme to the *cis* compartments of aerolysin nanopores. The locations of the peptides clusters used to determine the resolution of the nanopore are indicated by red (Lys4) and green (Lys5) arrows. The final concentration of trypsinated lysozyme was 10 ng/μL. Data were recorded in a mixture of 1 M KCl and 50 mM citric acid that was buffered to pH 3.0 using bis-tris propane at an applied potential of +150 mV.

Lowering the pH even more improved the resolution of Aer^K238D^, in particular for the small peptides with *I*_ex_% values between 20 and 40% (Figure 1F). The average dwell time also increased from 720 ± 50 ms at pH 3.8 to 990 ± 110 ms at pH 3.0. This effect might be partially electrostatic. The p*K*_a_ of residue Asp238 in Aer^K238D^ is approximately 3.9 (estimated using PROPKA3 software), indicating that at pH 3.8 approximately 50% of the seven aspartates in Aer^K238D^ will be negative charged, while at pH 3.0 the residues will be deprotonated about 10% of the time. Encouraged by the increased resolution of Aer^K238D^, we aimed to further improve the resolution with the addition of an aromatic residue directly above the aspartate, forming an acidic–aromatic sensing region. Our reasoning was that the acidic and aromatic residues would interact with the positively charged peptides through electrostatic and cation−π interactions, respectively. We introduced an aromatic residue at different positions within the barrel of Aer^K238D^ nanopores. We introduced phenylalanine residues instead of tryptophan, as the bulky tryptophan might lead to the frequent gating of the pore. In contrast, phenylalanine can be placed at different positions in the barrel without disrupting the pore while still providing significant aromaticity. The phenylalanine was introduced at four lumen-facing positions along the β-barrel, which were increasingly distant from the introduced acidic residue Asp238 (Figure 2A): Ala260Phe (0.4 nm from Asp 238), Ser264Phe (1.4 nm), Gln268Phe (2.4 nm), and Ser272Phe (3.4 nm). The phenylalanine mutants showed longer current blockades compared to that of Aer^K238D^ (Figure 2B) and largely better resolution. In fact, when the phenylalanine was placed 0.4 or 1.4 nm above the acidic residue, the average dwell time of the peptides increased by an order of magnitude compared to that of Aer^K238D^ (from 0.99 ± 0.11 ms in Aer^K238D^ to 12.8 ± 1.8 and 11.2 ± 2.7 in Aer^K238D+A260F^ and Aer^K238D+S264F^, respectively). The increased dwell time allows for a more accurate determination of the value of *I*_ex_%. In turn, the clusters of the peptides within the lysozyme spectrum became narrower (Figure 2C–F), indicating that more peptides can be identified simultaneously.

### Identification of Peptide Clusters Using the Event Noise

**Figure 3:**
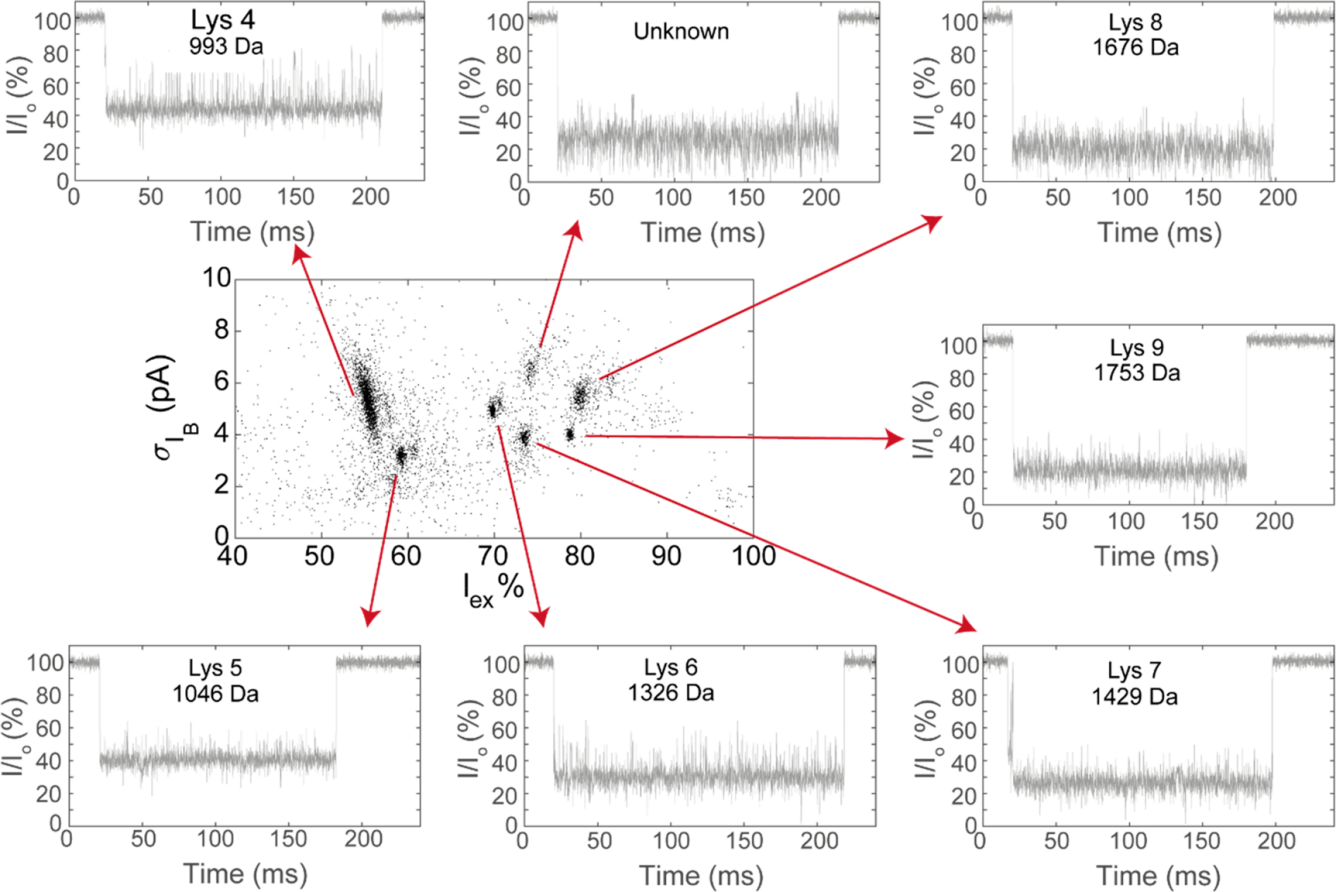
Detection of trypsinated lysozyme in Aer^K238D+A260F^. Plot of the event noise vs *I*_ex_% with a representative current blockade for each event. The plot shows the data from three nanopores combined after baseline correction. Data were recorded in a mixture of 1 M KCl and 50 mM citric acid that was buffered to pH 3.0 using bis-tris propane at an applied potential of +150 mV. The sequences of the peptides are as follows: Lys4, WWC_m_NDGR; Lys5, GTDVQAWIR; Lys6, GYSLGNWVC_m_AAK; Lys7, FESNFNTQATNR; Lys8, IVSDGNGMNAWVAWR; and Lys9, NTDGSTDYGILQINSR. C_m_ indicates a cysteine alkylated using iodoacetamide (IAA).

We noticed that the peptide current blockades of Aer^K238D+A260F^ showed different current fluctuation intensities. Therefore, we also calculated the event noise (*σ*_*I*_B__) from the standard deviation of the ionic current during the event. Rewardingly, the event noise of each cluster converged to a well-defined value with a narrow distribution (Figure 3A). Seven distinct peptide clusters could be detected compared to the five clusters observed in the *I*_ex_% versus dwell time analysis, specifically five clusters between 70 and 80 *I*_ex_% and two more between 50 and 60 *I*_ex_%. To assign the different peptides to current signals, we tested the nine synthetic peptides whose sequences corresponded to the peptides expected to be produced by the trypsination of lysozyme (Table S1). These peptides were named Lys1–Lys10 from the lowest to highest molecular weight. However, Lys10 (*M*_W_ = 2508 Da) was not tested because it could not be synthesized. Nonetheless, its large mass was outside the range of the peptides that are expected to be observed with aerolysin nanopores. Each cluster could be identified from the individual addition of the peptides to Aer^K238D+A260F^ (Figure S4). Seven peptides (Lys4–Lys9) were assigned. Interestingly no cluster was observed for Lys1–Lys3. Experiments with individual peptides revealed that Lys2 and Lys3 can be detected (Figure S5). Indeed, mass spectroscopy analysis (Table S2) revealed that Lys2 and Lys3 were present at low concentrations. Synthetic Lys1 (MW 517) did not induce blockades (Figure S5), indicating that it is probably too small to be detected by aerolysin. Interestingly, one of the clusters in the *σ*_*I*_B__ versus *I*_ex_% graph of trypsinated lysozyme remained unidentified. The latter peptide cluster might belong to deamidated Lys7, since the *I*_ex_% value is similar to that of Lys7 and some deamidated Lys7 was detected in the MS measurement of the trypsinated lysozyme (Table S2).

To quantify the resolutions of the different nanopores, we considered two event clusters that were visible in both the phenylalanine mutants corresponding to Lys4 (WWC_m_NDGR, *M*_w_ = 993 Da) and Lys5 (GTDVQAWIR, *M*_W_ = 1046 Da), two peptides that were present in high concentrations in the trypsinated lysozyme and differ in mass by 53 Da (Table S1). We calculated the mean (μ_*i*_) and standard deviation (σ_*i*_) for each cluster from Gaussian fittings to current histograms. The resolution *R*_s_ between the two clusters was then calculated as
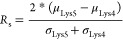
1where *R*_s_ should be at least 2 in order to completely separate the peaks.^23^ The two event clusters overlapped in Aer^K238D^ (Figure 1F) but could be distinguished in the phenylalanine mutants (Figure 2C–F and Table S3). Although the peaks were observed in all phenylalanine nanopores, the best resolution was observed when the distance between the acidic residue and the phenyl group was 1.4 nm. Interestingly, the introduction of aromatic residues 3.4 nm away from the acidic residue increased the spread of the event clusters despite inducing only slightly longer dwell times compared to those of Aer^K238D^, suggesting that the interaction between the acidic–aromatic constriction and the peptides is likely to have a role in improving the resolution. The voltage dependency with synthetic Lys4 and Lys5 peptides showed that the best resolution was at potentials higher than +125 mV (Figure S6), no large difference in resolution or dwell time was observed at potentials higher than +125 mV.

### CytK Engineering for Nanopore Peptide Spectroscopy

**Figure 4:**
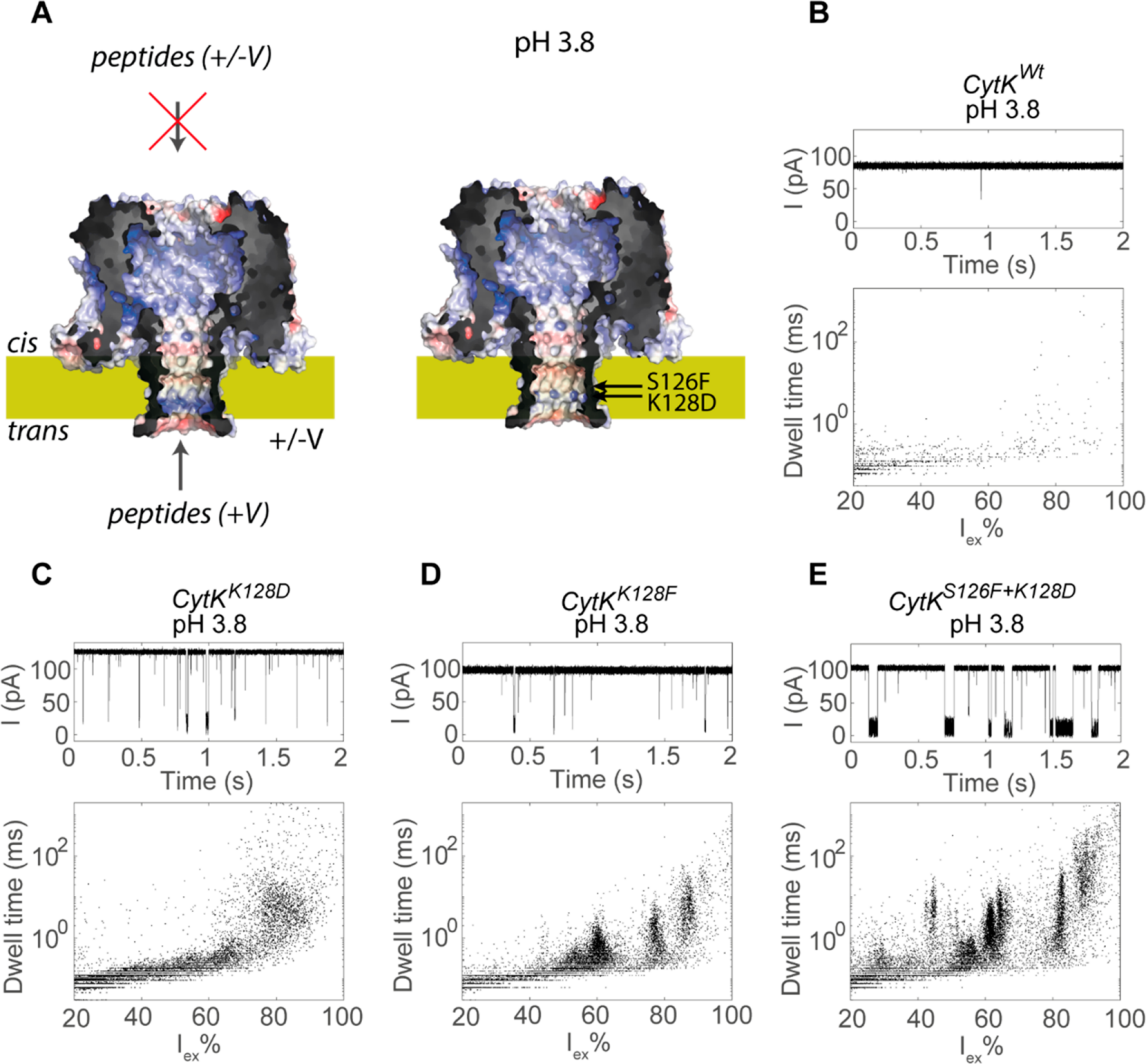
Detection of trypsinated lysozyme in CytK at pH 3.8. (A) Structure of CytK based on the homology model with αHL. The mutated residues Ser126 and Lys128 are indicated. The nanopores are shown as surface potential maps at pH 3.0 with the positively and negatively charged regions in blue and red, respectively. (B–E) Ionic current trace and event characteristics of CytK after the addition of 4 μg of trypsinated lysozyme in the *trans* chamber. The final concentration of trypsinated lysozyme was 10 ng/μL. Data were recorded in a mixture of 1 M KCl and 50 mM citric acid that was buffered to pH 3.8 using bis-tris propane at an applied potential of +100 mV.

Since the introduction of the acidic–aromatic sensing region greatly enhanced peptide detection in aerolysin nanopores, we tested this strategy with another β-barrel nanopore. We selected cytotoxin K (CytK). Although no crystal structure of CytK is available at present, the nanopore shares homology with β-PFTs such as α-hemolysin.^35^ We chose CytK instead of α-hemolysin because α-hemolysin was reported to show gating at low pH conditions.^36^ The conductance of CytK^Wt^ in 2 M KCl at pH 7.5 (2.08 ± 0.04 nS, calculated from linear regression of IV curves between +50 and −50 mV) was almost identical to that of α-hemolysin under the same conditions (2.08 nS),^37^ strongly suggesting that CytK also forms a heptameric nanopore. The structure of CytK was then made by homology modeling (Figures 4A and S7). We subsequently tested the ability of this nanopore to capture peptides from trypsinated lysozyme at low pH. The addition of the lysozyme peptides to either side of the nanopore at pH 3.8 (CytK^Wt^ did not form nanopores at pH 3.0) induced only very few events, indicating that the wild-type nanopore detected peptides poorly at low pH (Figure 4B). Therefore, we engineered the *trans* side of the β-barrel by introducing mutations that were similar to the Aer mutants using a predicted structure of CytK. We noted that CytK contains a lumen-facing lysine on the *trans* side of the β-barrel at position 128 (Figure 4A) and the substitution of this lysine by aspartate (CytK^K128D^) creates a sensing region similar to that of Aer^K238D^. CytK^K128D^ was indeed able to capture peptides (Figure 4C) but only when the peptides were added to the *trans* compartment. However, the events did not converge to detectable clusters (Figure 4C). As observed for aerolysin, the substitution of an aromatic residue (CytK^K128F^) improved the resolution of the peptides in the nanopore (Figure 4D), but only when the lumen-facing lysine was not present (i.e., CytK^S126F^ cannot detect peptides efficiently, Figure S8A). To combine an acidic–aromatic recognition element, thereby forming a sensing region similar to that of Aer^K238D+S260F^, we simultaneously substituted the lysine at position 128 with aspartate and introduced a phenylalanine 0.6 nm above the aspartate at position 126 (CytK^S126F+K128D^). As observed from the aerolysin nanopore, the double-mutant CytK^S126F+K128D^ increased the event duration, allowing for the detection of event clusters at both pH 3.8 (Figure 4E) and pH 3.0 (Figure S8B). The latter pH could be measured, as CytK^S126F+K128D^ inserted into lipid bilayers at lower pH values than CytK^Wt^. Contrary to the aerolysin mutants, the substitution of one amino acid dramatically changed the *IV* curves of the CytK mutants only if the mutation altered the charge in the barrel (e.g., CytK^K128D^ and CytK^K128F^, Figure S9).

### Mechanism of Peptide Capture and Translocation

To investigate the mechanism of peptide transport, we measured the voltage dependency of peptide transport. It is generally accepted that the decrease of a peptide’s dwell time with the applied potential is a strong indication that the analyte is translocating the nanopore.^38,39^ We measured two peptides (Lys5 (GTDVQAWIR) and Lys7 (FESNFNTQATNR)) that were also present in the trypsinated lysozyme (Table S2). These peptides were selected because they showed well-defined clusters in Aer^K238D+A260F^ (Figure 3A). In the aerolysin nanopore, the dwell time increased with the voltage for both peptides and reached a plateau for Lys5 at voltages above +130 mV, suggesting that the peptides might not translocate the nanopore. The dwell time started to decrease slightly at higher voltages, but measurements at potentials greater than +200 mV are challenging due to membrane instability. By contrast, the dwell time of both peptides in CytK decreases with the applied potential, suggesting that both peptides are likely to translocate the nanopore. Furthermore, despite both nanopores captured peptides at the positive bias, aerolysin nanopores captured peptides only from the *cis* side, while CytK capture peptides from the *trans* side, indicating that both the capture mechanism of and the transport of peptides in the two β-barrel nanopores are different. The transport mechanism across the two β-barrel nanopores might be understood by considering the forces acting on the peptides. At an acidic pH, the charge of peptides becomes more uniform than that at a physiological pH. This is because nearly all peptides carry a net positive charge corresponding to the N-terminus and another at the C-terminus given that trypsin cleaves after the K and R residues. At the same time, the negative charge normally carried by acidic residues is largely removed. As a result, the overwhelming majority of peptides will carry a positive charge and will experience an electrophoretic force (EF) toward the anode. Another force that influences the capture of peptides is the electro-osmotic flow (EOF),^21,40,41^ which is the directional motion of water across the nanopore in ion selective nanopores.

**Figure 5:**
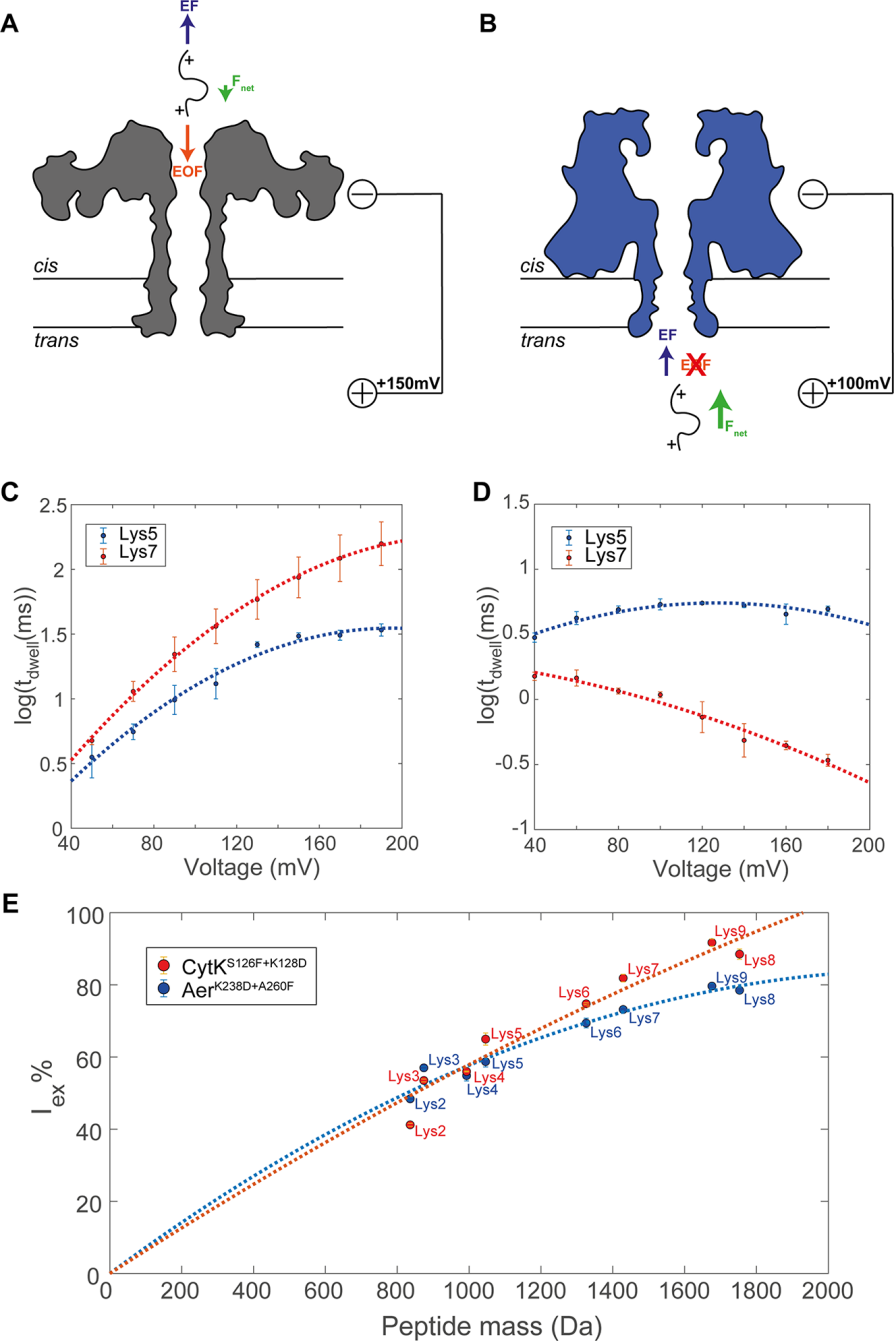
Mechanism of peptide capture and recognition by aerolysin and CytK nanopores. (A and B) Schematic illustration of the forces acting on the translocating peptide in Aer^K238D+A260F^ and CytK^S126F+K128D^, respectively, under a positive applied potential at low pH. EF indicates the electrophoretic force, EOF is the electro-osmotic flow, and *F*_net_ is the net force on the peptide. (C and D) Voltage dependency of the dwell time of peptides Lys5 (GTDVQAWIR) and Lys 7 (FESNFNTQATNR) in Aer^K238D+A260F^ and CytK^S126F+K128D^, respectively. The lines indicate a second-order polynomial fitting to the data. (E) Excluded current versus peptide mass with a second-order fit bound to [0,0] for Aer^K238D+A260F^ (blue) and CytK^S126F+K128D^ (red). The error bars refer to the standard error in the average value of (C and D) log_10_(dwell time) or (E) *I*_ex_% between three measurements in different nanopores.

To understand the direction of the EOF, we measured the ion selectivities of aerolysin and CytK nanopores with different charge distributions in the lumen at different pH values (Table S4). Aerolysin and CytK nanopores contain several charged residues in the lumen of the pore, located primarily at the ends of the β-barrel (Figure S10), that influence the ion selectivity of the pore. We found that Aer^Wt^ is anion-selective (K^+^/Cl^–^ selectivity ratio of 0.76 ± 0.07, Table S4) at pH 7.5, which is likely caused by the positively charged region on the *trans* side of the barrel (Figure 1A) and indicates that at positive applied potentials the EOF is from *cis* to *trans*. Lowering the pH of the solution increased the EOF (K^+^/Cl^–^ = 0.38 ± 0.03 at pH 3.8) due to increased positive charge of the nanopore surface (Table S4). The replacement of lysine with aspartate only slightly reduced the EOF (K^+^/Cl^–^ = 0.53 ± 0.03) at pH 3.8. Therefore, in aerolysin peptides are captured by the EOF against the applied potential (Figure 5A). This tug of war between the electrophoretic force and the EOF helps explain the long dwell times in Aer^K238D+A260F^ and the complex voltage dependency of Lys5 and Lys7 (Figure 5C). In the case of CytK, the nanopore is not ion-selective (K^+^/Cl^–^ = 1.02 ± 0.02) at pH 7.5 (Table S4), indicating that the EOF is weak or inexistent. Lowering the pH makes the nanopore anion-selective (K^+^/Cl^–^ = 0.58 ± 0.03 at pH 3.8). However, when Lys128 was substituted with aspartate the nanopore became slightly cation-selective at pH 3.8 (K^+^/Cl^–^ = 1.10 ± 0.05) and nonselective at pH 3.0 (K^+^/Cl^–^ = 0.99 ± 0.02) (Table S4). Hence, the electro-osmotic flow in CytK^K128D^ at pH 3.0 does not influence the capture of peptides, and the peptides are captured following the EF (Figure 5B). This is compatible with the relatively short dwell times, which reduce further as the voltage is increased (Figure 5D). Interestingly, the peptides are not captured from the *cis* side of CytK or the *trans* side of aerolysin. Most likely, this is due to the electrostatic energy barrier given by the positively charged residues at the *cis* entry of CytK and the *trans* entry of aerolysin.

Using the synthetic lysozyme peptides (Lys2–Lys9), we correlated the value of *I*_ex_% with the MW in Aer^K238D+A260F^ and CytK^S126F+K128D^. We generally observe an increase in the value of *I*_ex_% with the increasing mass of the peptides (Figure 5E), but some discrepancies were observed. Peptide Lys9 has a higher mass than peptide Lys8 but has a lower excluded current for both nanopores. Similarly, peptide Lys2 has a significantly lower *I*_ex_% than Lys3 despite Lys2 having a higher mass (due to the alkylation of the cysteine). These discrepancies could be explained by considering that *I*_ex_% reflects the excluded volume rather than the mass of the peptide.^42,43^

## Conclusions

Biological nanopores are emerging as sensors capable of detecting peptides and proteins at the single-molecule level. Recently, we showed that proteins lysed by a specific protease such as trypsin might be recognized in bulk using α-helical FraC nanopores.^19^ We also showed that a proteasome can be introduced directly above a β-barrel nanopore.^18^ Therefore, if β-barrel nanopores can be used to detect peptides, this system might be used for the single-molecule identification of proteins. In this work, we investigated the ability of two β-barrel nanopores to identify peptides from the protease digestion of lysozyme. We found that neither the wild-type aerolysin nanopores nor the wild-type CytK nanopores could efficiently detect the peptides at pH 7 or 3. Interestingly, a work appeared during the peer review of this work showing that lysozyme-digested peptides can be observed using an Aer^Wt^ nanopore at pH 7.5 under 3.6/4 M KCl *cis*/*trans* ionic conditions,^44^ respectively, suggesting that electrostatic interactions might play a role in either the entry of peptides into the nanopore or the retention of peptides inside the nanopore. Both aerolysin and CytK nanopores have a lysine residue near the *trans* entry of the nanopore, and its substitution with an acidic residue improved the entry of peptides into the nanopore. However, the further introduction of an aromatic amino acid was crucial to distinguish among trypsinated peptides. Scanning a phenylalanine along the barrel of aerolysin revealed that more efficient recognition occurred when the acidic and aromatic residues are in close vicinity (i.e., within 0.4–1.4 nm).

Our work also revealed information on the mechanism of peptide entry and translocation. In the aerolysin nanopore, the EOF induces peptide capture even against the electrophoretic force. This is an important finding because in nanopore protein sequencing it might be necessary to transport polypeptides across a nanopore against the applied potential. For peptides, the resolution was determined by the distance between the aromatic residue and the acidic residue. When phenylalanine was placed directly above the aspartic acid, the events converged to well-defined clusters, whereas a larger distance between the acidic and aromatic residue decreased the resolution of the nanopore. Interestingly, one mutant, Aer^K238D+A260F^, could accurately distinguish between two similar peptides based on their event noise, suggesting that they interact differently with the sensing region of the nanopore. This finding is important because it provides another dimension to identify peptides that does not only rely on the volume or mass of the peptide but instead on the chemical properties of the peptide. This is advantageous for measurements in complex samples.

In CytK, the acidic–aromatic recognition site was also important for peptide capture. However, we found that entry into the nanopore was facilitated by the EF rather than the EOF. This mechanism is similar to the entry of peptides into an α-helical FraC nanopore where an aromatic sensing region was introduced to improve peptide recognition, and peptides enter the nanopores by the action of the EF rather than EOF forces. The advantageous effect of the acidic–aromatic sensing region on the resolution of the peptides might originate from increased analyte–nanopore interactions. Peptides that translocate the nanopore are most likely detected when they interact with the nanopore surface, as free translocation would be too quick. The acidic–aromatic sensing region may interact with the peptide via both electrostatic and cation−π interactions, thus increasing the likelihood of peptides to be sufficiently interrogated to accurately determine the ionic current blockade. Interestingly, the CytK mutant with an acidic–aromatic sensing region can detect small lysozyme peptides (Lys2 and Lys3) more accurately than the aerolysin mutants despite showing overlap in event clusters for large peptides (Lys6–Lys9 with *I*_ex_% = 80–100%). Most likely, the larger peptides have long dwell times in aerolysin due to the tug of war between the EF and EOF, but shorter peptides translocate too fast to be accurately detected. CytK, in contrast, has relatively short dwell times for the lysozyme peptides due to the lack of an EOF, but shorter peptides dwell longer.

## Materials and Methods

### Chemicals

Potassium chloride (>99.5%), sodium chloride (>99.5%), magnesium chloride (anhydrous, >98.5%), imidazole (>99%), LB medium, 2YT medium, dodecyl-β-d-maltosid (DDM, >99%), 1,4-dithiothreit (DTT), d(+)-glucose, Tris (Pufferan, >99.9%), and lysozyme (albumin-free) were purchased from Carl Roth. Citric Aacid (anhydrous, 99.6%) was purchased from Acros Organics. Bis-tris propane (>99%), pentane (>99%), *n*-hexadecane (>99%), and iodoacetamide (IAA) were purchased from Sigma. Ampicillin sodium salt, isopropyl-β-d-thiogalactopyranoside (IPTG), and trypsin (from porcine pancreas) were obtained from Thermo Fisher Scientific. 1,2-Diphytanol-*sn*-glycero-3-phosphocholine (DPhPC) was obtained from Avanti Lipids. Synthetic lysozyme peptides (Lys1, Lys2, Lys3, Lys4, Lys5, Lys6, Lys7, Lys8, and Lys9) were synthesized by GenScript.

### Aerolysin and CytK Plasmid Construction

The genes for (pro-)aerolysin (*Aeromonas hydrophila*, P09167) and CytK (*Bacillus cereus*, Q09KJ1) were synthesized by Integrated DNA Technologies and ligated into the pT-SC7 plasmid. The DNA and amino acid sequences of both genes can be found in the Supporting Information.

### Mutagenesis of Aerolysin and CytK

Site-directed mutagenesis was performed by amplifying part of the gene by PCR with *Taq*-polymerase (REDTaq DNA Polymerase, Sigma) using a forward primer containing the desired mutation and the T7-reverse primer (Table S7). The product, a megaprimer, was subsequently used to amplify the whole plasmid in a second round of PCR using the *Phire* Hot Start II polymerase (Thermo Fisher). The product of the second PCR was digested with DpnI (FastDigest DpnI, Thermo Fisher) for 30 min at 37 °C. For plasmid amplification, 1 μL of the DpnI-digested PCR mixture was transformed into electrocompetent *E. Cloni* cells. The transformed cells were grown on LB agar plates supplemented with 1% glucose and 100 μg/mL ampicillin. The next day, colonies were inoculated in 5 mL of LB medium supplemented with 100 μg/mL ampicillin, then grown overnight at 37 °C. The cells were pelleted by centrifugation at 4000 rpm for 5 min, and the plasmid was purified using a GeneJET Plasmid MiniPrep kit (Thermo Fisher). The purified plasmid was sequenced to confirm the presence of the desired mutation.

### Expression and Purification of Pro-Aerolysin

The plasmid containing the pro-aerolysin gene was transformed into *Escherichia coli* BL21(DE3) cells using electroporation. The transformed cells were grown overnight at 37 °C on LB agar plates supplemented with 1% glucose and 100 μg/mL ampicillin. On the next day, the colonies were resuspended and grown in 200 mL of the 2YT medium at 37 °C until the OD_600_ reached 0.6–0.8. At this point, the expression was induced by the addition of 0.5 mM IPTG. The culture was incubated overnight at 25 °C. Afterward, the cells were pelleted by centrifugation at 4000 rpm for 15 min. The cell pellets were stored at −80 °C for at least 30 min. Cell pellets of the 100 mL culture were thawed and resuspended in 20 mL of lysis buffer, containing 150 mM NaCl, 20 mM imidazole, and 15 mM Tris buffered to pH 7.5 supplemented with 1 mM MgCl_2_, 0.2 units/mL DNaseI (Thermo Fisher), and approximately 1 mg of lysozyme. The mixture was incubated for 30 min at RT and then sonicated using a Branson Sonifier 450 (2 min, duty cycle 30%, output control 3) to fully disrupt the cells. Cell debris was pelleted by centrifugation at 6000 rpm for 20 min, and the supernatant was transferred to a fresh Falcon tube. Meanwhile, 200 μL of the Ni-NTA bead solution (Ni-NTA agarose, Qiagen) was washed with a wash buffer containing 150 mM NaCl, 20 mM imidazole, and 15 mM Tris buffered to pH 7.5. The beads were added to the supernatant and incubated at RT for 5 min under constant rotation. Afterward, the solution was loaded on a Micro Bio-Spin column (Bio-Rad) and subsequently washed with 5 mL of the wash buffer. The bound protein was eluted in fractions of 200 μL using an elution buffer (150 mM NaCl, 300 mM imidazole, and 15 mM Tris buffered at pH 7.5). The pro-aerolysin fractions can be stored at 4 °C for several weeks.

### Oligormerisation of Aerolysin

Pro-aerolysin was incubated with trypsin in a 1:1000 trypsin/protein mass ratio for 15 min at room temperature. Trypsin cleaves off the C-terminal pro-peptide (residues 446–493), resulting in aerolysin monomers that spontaneously assemble into heptameric nanopores. Afterward, the trypsinisation reaction could be quenched by the addition of 0.01 M HCl. Aerolysin nanopores can be stored at 4 °C for several weeks.

### Purification of CytK Nanopores

The plasmid containing the CytK gene was transformed into *E. coli* BL21(DE3) cells using electroporation. The transformed cells were grown overnight at 37 °C on LB agar plates supplemented with 1% glucose and 100 μg/mL ampicillin. The next day, the colonies were resuspended and grown in 200 mL of the 2YT medium at 37 °C until the OD_600_ reached 0.6–0.8. At this point, the expression was induced by the addition of 0.5 mM IPTG, and the culture was incubated overnight at 25 °C. Afterward, the cells were pelleted by centrifugation at 4000 rpm for 15 min, and the cell pellets were stored at −80 °C for at least 30 min. Cell pellets of the 200 mL culture were resuspended in 20 mL of lysis buffer containing 150 mM NaCl, 20 mM imidazole, and 15 mM Tris buffered to pH 7.5 supplemented with 0.02% DDM, 1 mM MgCl_2_, 0.2 units/mL DNase1, and approximately 1 mg of lysozyme. The mixture was incubated for 30 min at RT and then sonicated using a Branson Sonifier 450 (2 min, duty cycle 30%, output control 3) to fully disrupt the cells. Cell debris was pelleted by centrifugation at 6000 rpm for 20 min, and the supernatant is transferred to a fresh falcon tube. Meanwhile, 200 μL of the Ni-NTA bead solution (Ni-NTA agarose, Qiagen) is washed with a wash buffer containing 150 mM NaCl, 20 mM imidazole, and 15 mM Tris buffered to pH 7.5 supplemented with 0.02% DDM. The beads were added to the supernatant and incubated at RT for 5 min. Afterward, the solution was loaded on a Micro Bio-Spin column (Bio-Rad) and subsequently washed with 5 mL of the wash buffer. The bound nanopores can be eluted in fractions of 200 μL using an elution buffer (150 mM NaCl, 300 mM imidazole, and 15 mM Tris buffered at pH 7.5 supplemented with 0.02% DDM). The CytK fractions can be stored at 4 °C for several months.

### Planar Lipid Bilayer Recordings

Nanopore recordings were performed using a chamber consisting of two compartments separated by a 25 μm thick Teflon (Goodfellow Cambridge Ltd.) membrane, which contained an aperture with a diameter of approximately 100 μm. Lipid membranes were formed by first applying 5 μL of 5% hexadecane in pentane near the aperture. The pentane was left to dry, and 400 μL of buffer (1 M KCl and 50 mM citric acid, titrated with either bis-tris propane to pH 3.0 or 3.8 or 1 M KCl with 50 mM Tris buffered at pH 7.5) was added to both compartments. Then, 10 μL of a 10 mg/mL solution of DPhPC dissolved in pentane was added on top of the buffer on each side of the chamber. The chamber was briefly left to dry to allow the evaporation of pentane. Ag/AgCl electrodes were inserted into to each compartment. The **cis** compartment was connected to the ground, and the *trans* electrode functioned as the working electrode. Planar lipid bilayers were formed by repeatedly lowering and raising the buffer reservoirs until a stable bilayer with a capacitance of approximately 100 pF was formed.

### Digestion of Lysozyme Using Trypsin

In 90 μL of buffer containing 150 mM NaCl and 50 mM Tris buffered to pH 7.5 was dissolved 100 μg of lysozyme (*Gallus gallus*). The protein contained cysteine residues that were reduced and alkylated to prevent disulfide bond formation from interfering with the digestion. To this end, we added 3 μL of 200 mM DTT to the mixture. We incubated the sample at 37 °C for 15 min to reduce the cysteines and then at 95 °C for 15 min to denature the protein. Afterward, the cysteine residues were alkylated by the addition of 7 μL of 200 mM IAA, followed by a 15 min incubation in the dark. The alkylated protein was digested overnight using a Trypsin Singles proteomics-grade kit (Sigma-Aldrich) in a 1:50 trypsin/protein mass ratio. The digested sample was stored at −20 °C until use. The sample was measured with ESI-MS to check the digestion and alkylation of the sample.

### Detection of Trypsinated Lysozyme in Aerolysin Nanopores

Approximately 1 μg of aerolysin was added to the *cis* chamber, and the bilayer was broken and reformed until a single channel was inserted into the bilayer. The orientation of the nanopore could be detected by a small asymmetry in the current–voltage characteristics of the nanopore. Before each recording, a 2 min blank was recorded at an applied potential of +150 mV. Afterward, 4 μL of trypsin-digested lysozyme was added to the *cis* compartment of the chamber. The analyte was measured for at least 10 min at an applied potential of +150 mV.

### Detection of Trypsinated Lysozyme in CytK Nanopores

A tiny amount of CytK (typically less than 1 ng) was added to the *cis* chamber, and the bilayer was broken and reformed until a single channel was inserted into the bilayer. The orientation of the nanopore could be detected by the asymmetry in the current–voltage characteristics of the nanopore. First, a 2 min blank was recorded at an applied potential of +100 mV. Afterward, 4 μL of trypsin-digested lysozyme was added to the *trans* compartment of the chamber. The analyte was measured for at least 10 min at an applied potential of +100 mV.

### Data Acquisition

The ionic current traces were recorded using a Digidata 1440A (Molecular Devices) instrument connected to an Axopatch 200B amplifier (Molecular Devices). All measurements were recorded with a sampling frequency of 50 kHz and a Bessel filter of 10 kHz. An additional 5 kHz digital Gaussian low-pass filter was applied prior to the event detection.

### Event Detection

First, we determined the open nanopore current (*I*_0_) and the noise in the open nanopore current (σ_*I*_0__) of the ionic current trace. To this end, we used Clampfit software to take the histogram of the ionic current and apply a Gaussian fit around the open nanopore current. *I*_0_ was determined from the center of the peak, and σ_*I*_0__ was determined from the standard deviation of the peak. Then, we detected events using a threshold search with a threshold of 5σ_*I*_0__ and with a minimum duration of 50 μs. The excluded current percent (*I*_ex_%) was calculated using , where ΔI_B_ is the magnitude of the current blockade.

### Reversal Potential Measurements

During reversal potential measurements, the electrodes were not in direct contact with the buffer solution but were connected via agarose bridges containing 2.5% agarose in a saturated KCl solution.^45^ Both compartments were filled with 400 μL of buffer, with 2 M KCl buffered to the desired pH using either Tris (pH 7.5) or citric acid and bis-tris propane (pH 3.0 and 3.8). The *IV* curve was measured under these symmetrical salt conditions between −100 and +100 mV in steps of 5 mV. Afterward, the concentration of KCl in the *trans* compartment was decreased to 0.5 M by replacing 300 μL of the buffer with a buffer without KCl and the subsequent perfusion of the compartment with the 0.5 M KCl buffer. The *IV* curve was measured between −100 and +100 mV in steps of 5 mV, and the reversal potential (*V*_r_) was estimated by linear regression of the curve between −20 and +20 mV. The ion selectivity (*P*_K_^+^/*P*_Cl_^–^) of the nanopore was calculated using
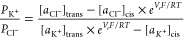
Where [*a*_Cl^–^/K^+^_] is the activity of K^+^ or Cl^–^ in the or *cis* or *trans* compartment, *F* is the Faraday constant (96 485 C/mol), *R* is the gas constant (8.3145 J/mol·K), and *T* the temperature (298 K). The activity was calculated by multiplying the concentration by the mean activity coefficient (0.649 for 0.5 M KCl and 0.573 for 2 M KCl).^46^ The values in Table S4 are the average and the standard deviation of three measurements in different nanopores.

### Structure Prediction CytK

An initial single-chain prediction of the CytK structure was made by homology modeling using the SWISS-MODEL prediction server.^47^ The structure of α-hemolysin (PBD 3ANZ)^48^ was used as a template. The predicted single chain was then superimposed on the remaining six chains of α-hemolysin using the open source version of PyMOL to create the heptameric structure.

### Calculation of Nanopore Electrostatics

Mutants of aerolysin and CytK were generated using PyMOL,^49^ after which the side-chains were relaxed in a vacuum for 5 ps with backbone restraints using GROMACS^50^ in combination with the AMBER99sb-ildn force field.^51^ The aerolysin and CytK models were processed using PDB 2pqr, which assigns partial charges to the atoms.^52^ The p*K*_a_ values of titratable side-chains were estimated using PROPKA3.^53^ Electrostatic maps were then calculated using APBS at the appropriate ion concentration and loaded in PyMOL as a color gradient for visualization.^54^ The total charge of the barrel lumen was estimated by considering individual residues and summing all partial charges of the residues with inward pointing side-chains, as calculated at the indicated pH by PROPKA3.

## Supporting Information Available

Measurements in other aerolysin and CytK mutants, CytK homology model, mass spectrometry measurements of trypsinated lysozyme, reversal potential measurements, and the DNA and corresponding amino acid sequences of Aer^Wt^ and CytK^Wt^ (PDF)

## Notes

The authors declare that the data supporting the findings of this study are available within the article and its Supporting Information or from the corresponding authors upon reasonable request.
