# Supplementary material for: β-Barrel Nanopores with an Acidic–Aromatic Sensing Region Identify Proteinogenic Peptides at Low pH
Source: ACS Nano. 2022 Mar 18;16(5):7258–68. doi: 10.1021/acsnano.1c11455 (PMC9134492; doi:10.1021/acsnano.1c11455)
Supplement: Supplementary file 1 — nn1c11455_si_001.pdf [file nn1c11455_si_001.pdf]

β-barrel Nanopores with an Acidic-Aromatic Sensing Region identify Proteinogenic Peptides at low pH

Roderick Corstiaan Abraham Versloot[1], Sabine Angenieta Paulina Straathof[1], Gemma Stouwie[1], Matthijs Jonathan Tadema[1], Giovanni Maglia[1]*

[1] Groningen Biomolecular Sciences and Biotechnology Institute, University of Groningen, Groningen, 9747AG, Netherlands

* Correspondence: g.maglia@rug.nl

Table of content

Figure S1: Additional ionic current traces of aerolysin nanopores P.3

Figure S2: Ionic current traces and event characteristics of Aer^{K238Q} and Aer^{K238F} P.4

Figure S3: IV curves of aerolysin Lys238 mutants. P.5

Figure S4: Measurement of lysozyme digest and individual peptides in the same AerK238D+A260F nanopore. P.6

Figure S5: Detection of small peptides P.7

Figure S6: Voltage dependency of the resolution between Lys4 and Lys5 P.8

in AerK238+S264F

Figure S7: Homology model for the structure of CytK P.9

Figure S8: Ionic current traces and event characteristics of $CytK^{S126F}$, and $CytK^{S126F+K128D}$ at pH 3.0 P.10

Figure S9: IV curves CytK mutants P.11

Figure S10: Schematic illustration of the positions of charged residues in the lumen of the nanopore P.12

Table S1: Expected peptide fragments after tryptic digestion of Lysozyme P.13

Table S2: Mass-spectrometry measurement of trypsinated lysozyme P.13

Table S3: Resolution of peptides in the phenylalanine mutants P.14

Table S4: Reversal potential measurements of aerolysin and CytK nanopores P.14

Table S5: Ionic current of aerolysin mutants in 1M KCl at pH 3.8 P.15

Table S6: Ionic current of CytK mutants in 1M KCl at pH 3.8 P.16

Amino acid sequence and DNA sequence of pro-AerWt and $CytK^{Wt}$ P.17

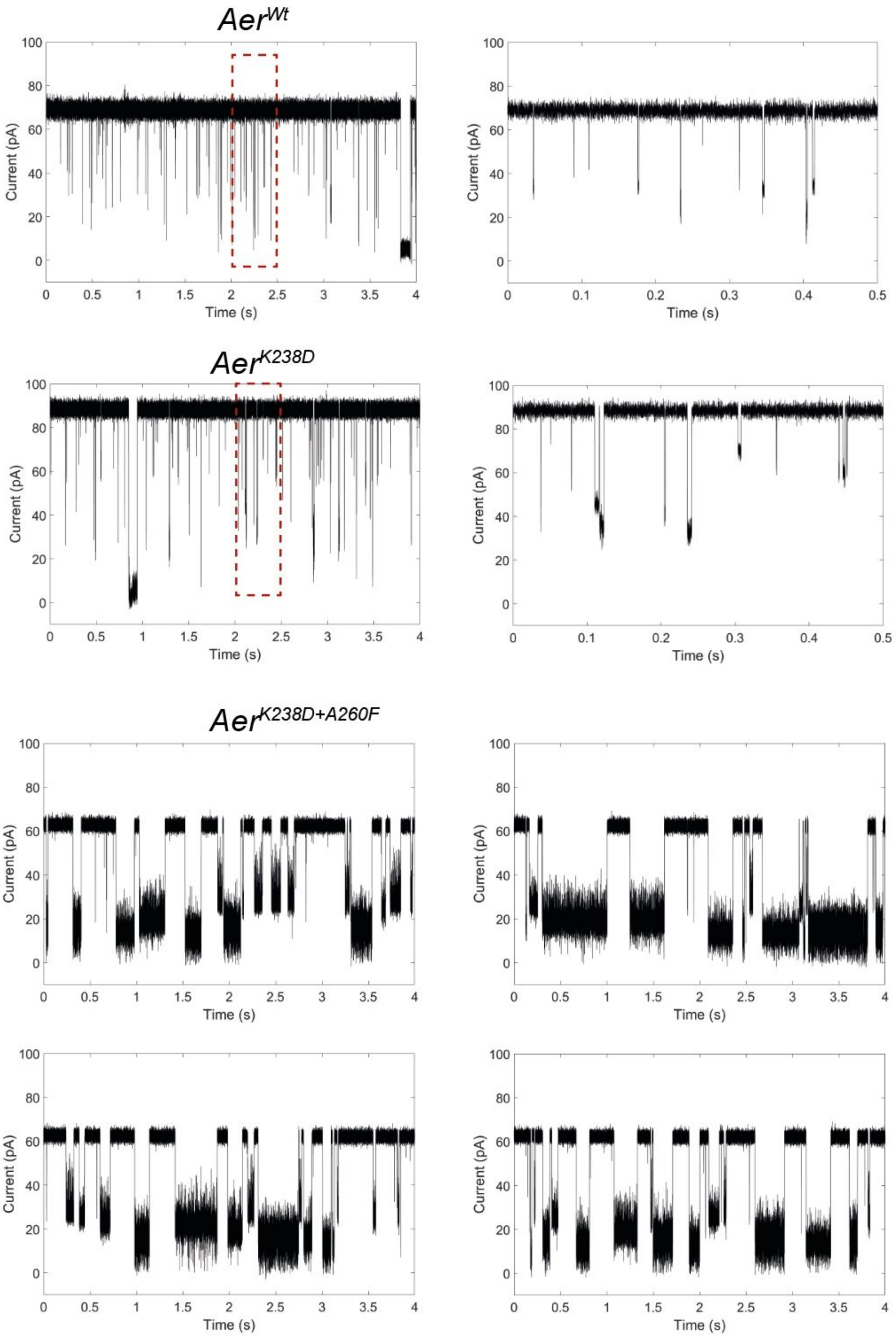

Figure S1: Additional ionic current traces of aerolysin nanopores. Events in aerolysin nanopores after the addition of 10 ng/µl of trypsinated lysozyme. For Aer^{Wt} (pH 3.8) and $\text{Aer}^{\text{K238D}}$ (pH 3.0) the right current trace is an expansion of the left trace as indicated. For $\text{Aer}^{\text{K238D+A60F}}$(pH 3.0), four different current traces are indicated. Data recorded in 1 M KCl, 50 mM citric acid, buffered to the final pH value using bis-tris propane at an applied potential of +150 mV. An additional 5 kHz Bessel filter was applied to the data shown in this figure.

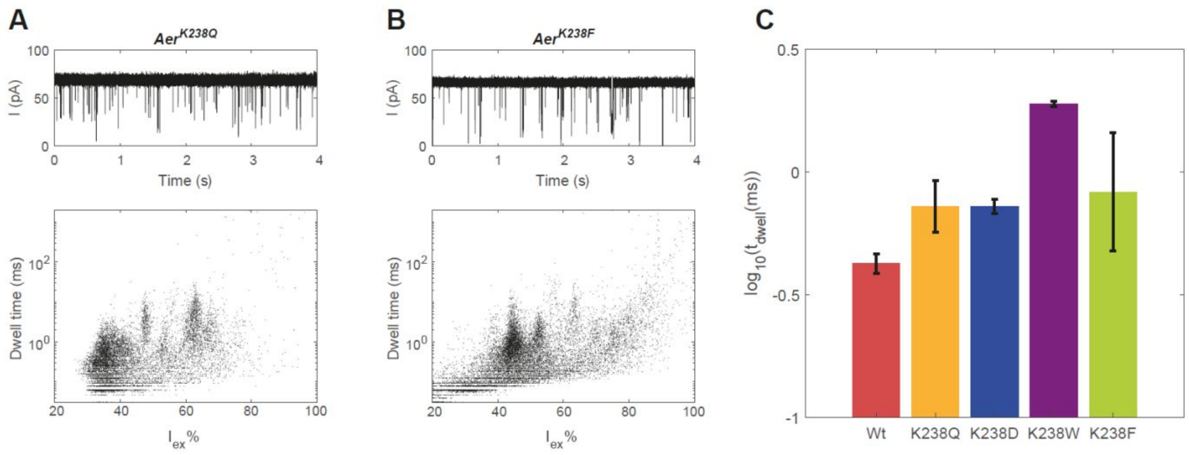

Figure S2: Ionic current traces and event characteristics of (A) Aer^{K238Q} and (B) Aer^{K238F} after the addition of 4 µg trypsinated lysozyme in the *cis* compartment. The final concentration of trypsinated lysozyme is 10 ng/µl. Data recorded at +150 mV applied potential in 1 M KCl and 50 mM citric acid, buffered to pH 3.8 using bis-tris propane. (C) Average dwell times of Aer^{Wt} and Lys238 mutants. Error bars indicate the standard deviation between three measurements in three different pores.

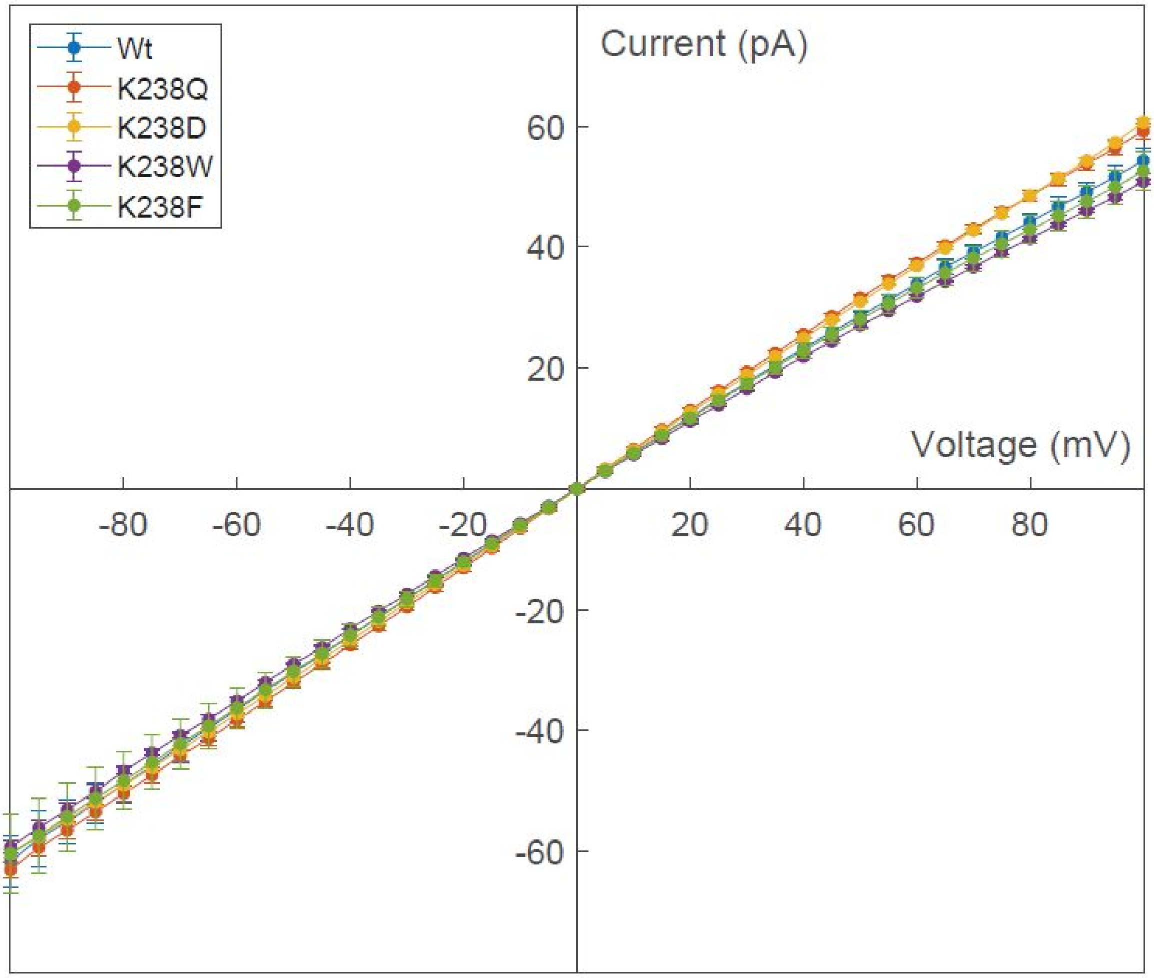

Figure S3: IV curves of aerolysin Lys238 mutants. Data recorded in 1 M KCl and 50 mM citric acid, buffered to pH 3.8 using bis-tris propane. Error bars indicate the standard deviation between measurements in at least three different nanopores.

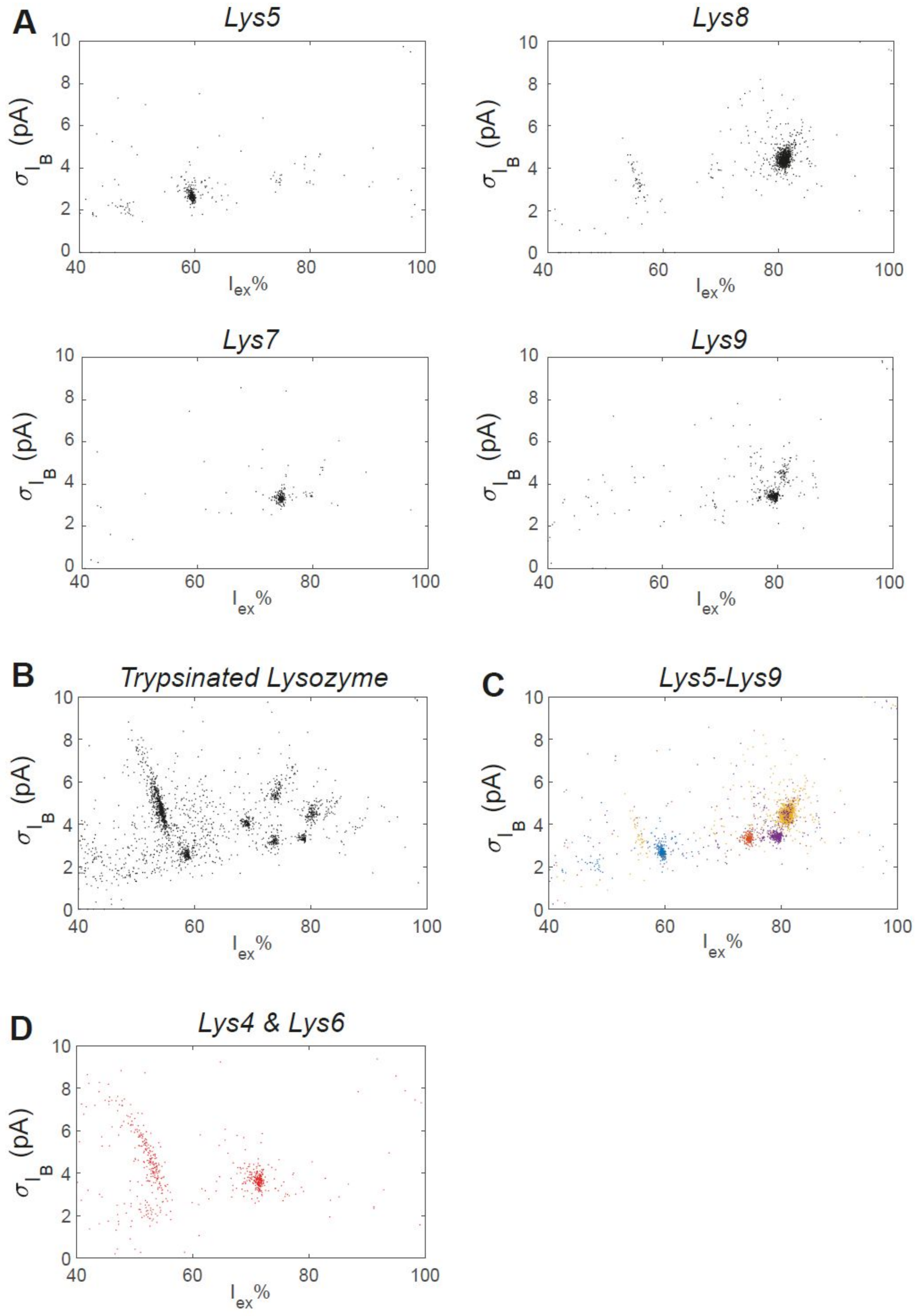

Figure S4: Measurement of lysozyme digest and individual synthetic peptides in the same $Aer^{K238D+A260F}$ nanopore. (A) nanopore spectrum of synthetic peptides. The chamber was perfused with fresh buffer in between the measurements of the lysozyme digest and each individual peptide. The measurement of Lys9 contained a trace amount of Lys8, because not all peptide was perfused out of the chamber. (B) The same nanopore was then used to test the spectrum of trypsinated lysozyme. (C) nanopore spectrum of the synthetic peptides: lys5 (blue), lys7 (orange), lys8 (yellow) and lys9 (purple). (D) Peptides Lys 4+6 measured in a different nanopore.

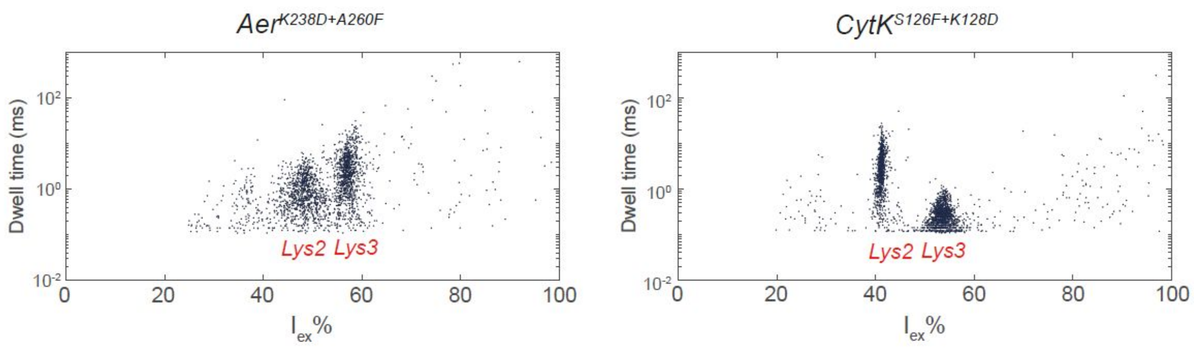

Figure S5: Detection of small peptides. Nanopore spectrum after the addition of 10 µM Lys1, Lys2 and Lys3 to Aer$^{K238D+A260F}$ (left) and CytK$^{S126F+K128D}$ nanopores. Data recorded at +150 mV applied potential (aerolysin) or +100 mV applied potential (CytK) in 1 M KCl and 50 mM citric acid, buffered to pH 3.0 using bis-tris propane.

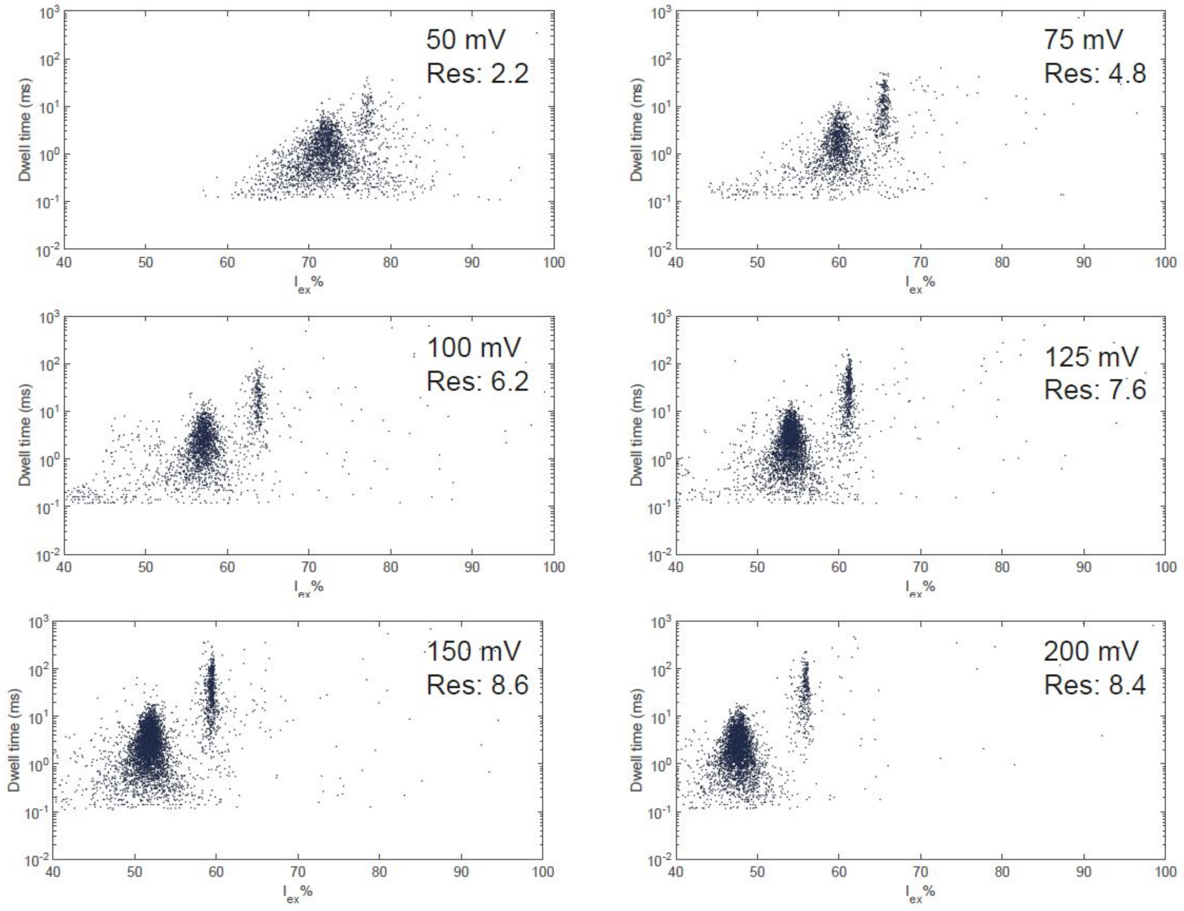

Figure S6: Voltage dependency of the resolution between Lys4 and Lys5 in $Aer^{K238+S264F}$. The figure shows the nanopore spectra after addition of a mixture of 2.5 µM Lys4 and 5 µM Lys5 to the *cis* chamber. Data recorded at +150 mV applied potential in 1 M KCl and 50 mM citric acid, buffered to 3.0 using bis-tris propane.

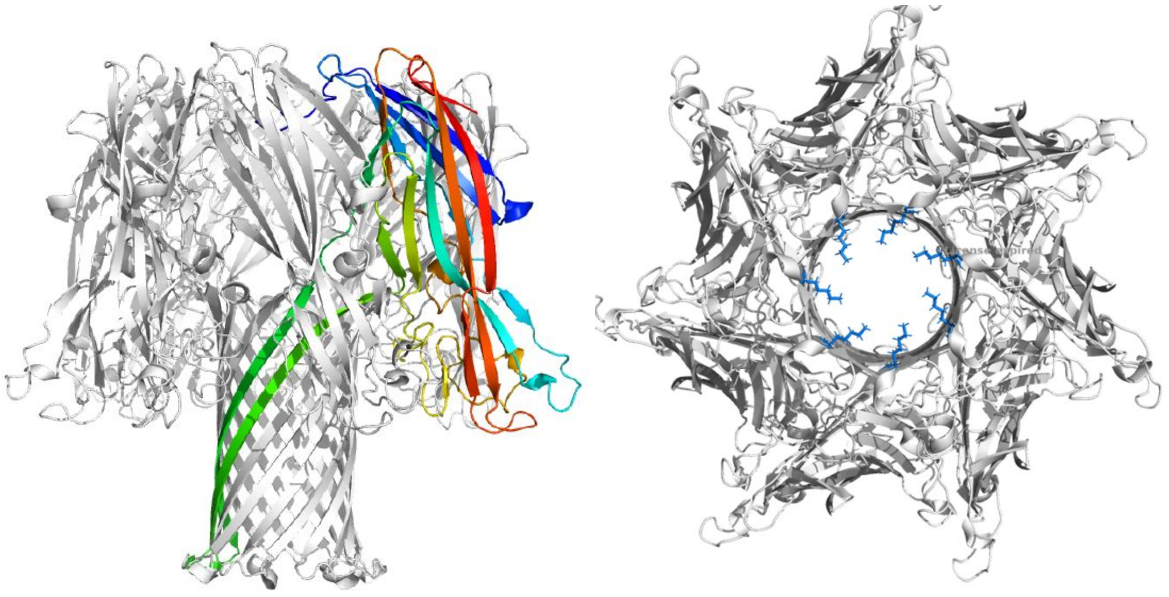

Figure S7: Homology model for the structure of CytK (left) and location of residue Lys128 (colored blue) at the bottom of the barrel (right)

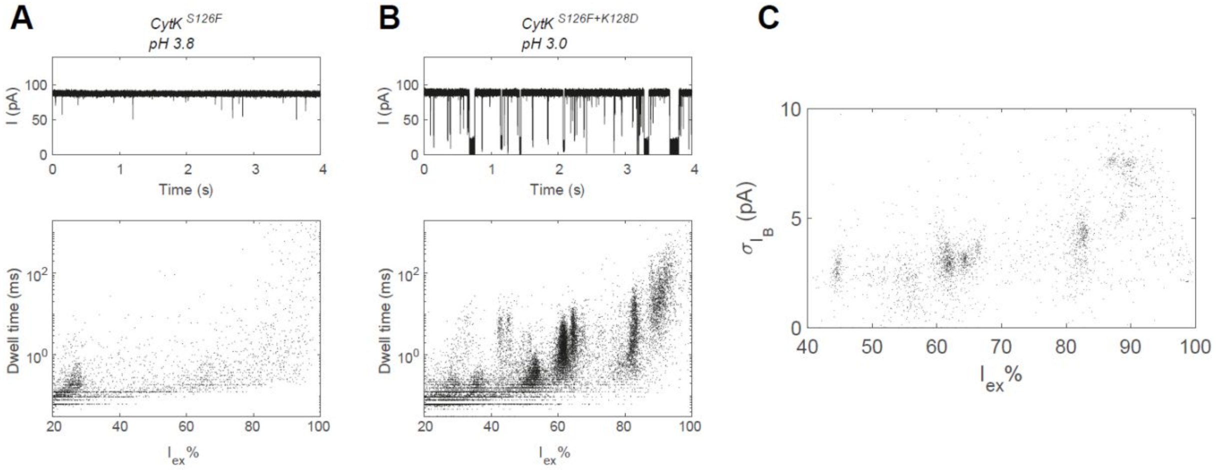

Figure S8: Ionic current traces and event characteristics of (A) CytK^{S126F} at pH 3.8 and (B) CytK$^{S126F+K128D}$ at pH 3.0 after the addition of 4 µg trypsinated lysozyme in the *trans* compartment. The final concentration of trypsinated lysozyme is 10 ng/µl. (C) I_{ex}% vs event noise (σ_{IB}) plot of the events detected in CytK$^{S126+K128D}$ after the addition of 4 µg trypsinated lysozyme in the *cis* compartment at pH 3.8. Data recorded at 150 mV applied potential in 1 M KCl and 50 mM citric acid, buffered to pH 3.8 or 3.0 using bis-tris propane.

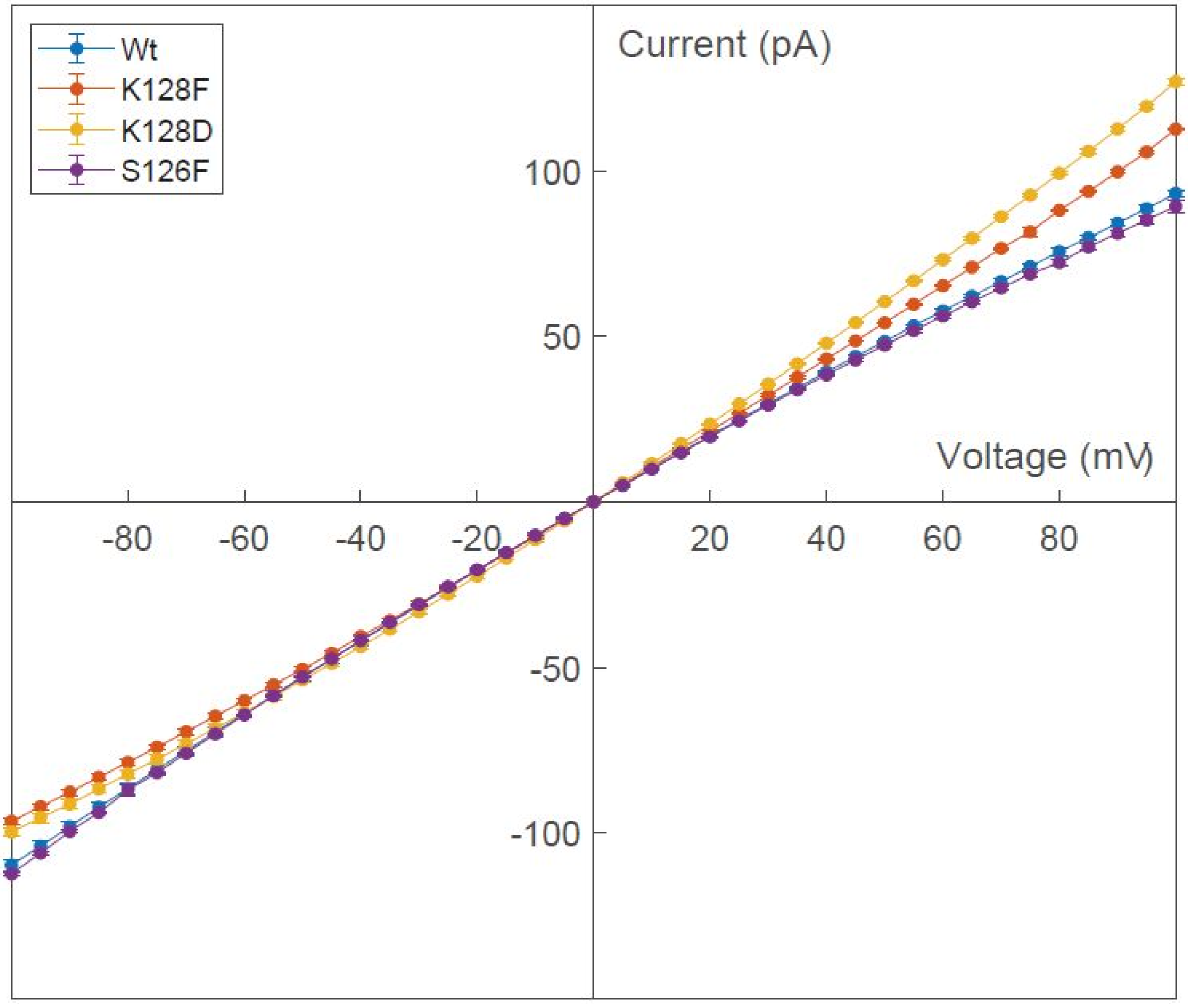

Figure S9: IV curves CytK mutants. Data recorded in 1 M KCl and 50 mM citric acid, buffered to pH 3.8 using bis-tris propane. Error bars indicate the standard deviation between measurements in at least three different nanopores.

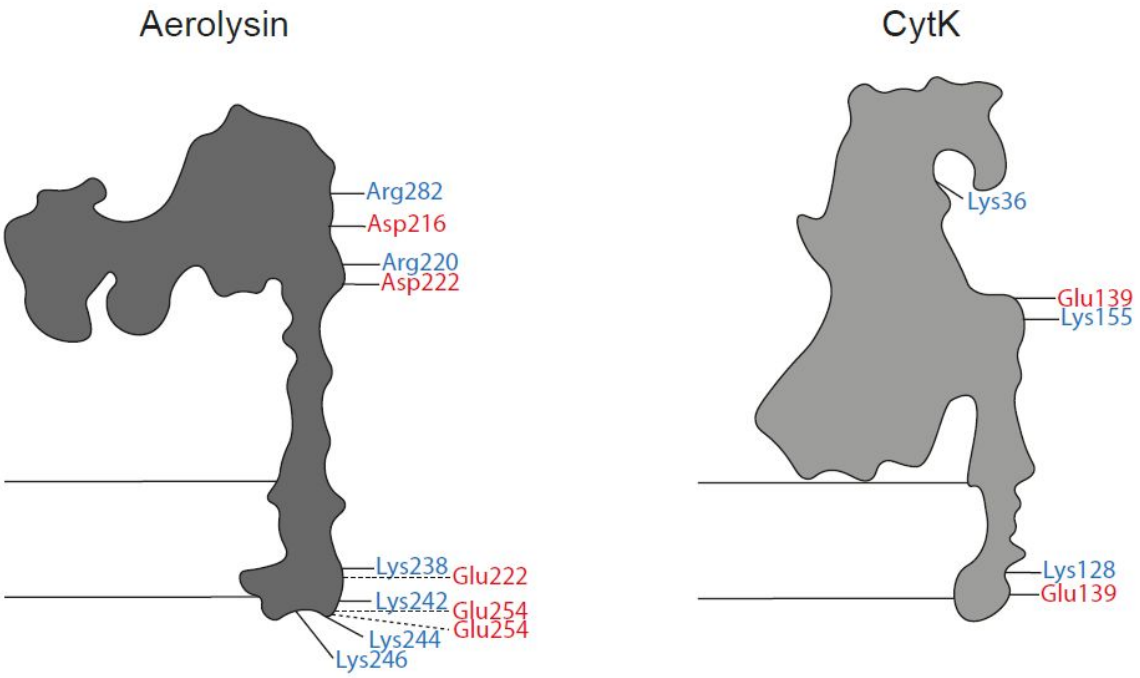

Figure S10: Schematic illustration of the positions of charged residues in the lumen of the Aerolysin (left) and CytK (right) nanopore.

Name	Peptide sequence	Position in the protein	Mass (Da)*
Lys10	NLC$_m$NIPC$_m$ SALLSSDITASVNC$_m$AK	92-114	2508.19
**Lys9**	**NTDGSTDYGILQINSR**	**64-79**	**1753.84**
**Lys8**	**IVSDGNGMNAWVAWR**	**116-130**	**1675.80**
**Lys7**	**FESNFNTQATNR**	**52-63**	**1428.65**
**Lys6**	**GYSLGNWVC$_m$AAK**	**40-51**	**1325.63**
**Lys5**	**GTDVQAWIR**	**135-143**	**1045.54**
**Lys4**	**WWC$_m$NDGR**	**80-86**	**993.39**
**Lys3**	**HGLDNYR**	**33-39**	**874.42**
**Lys2**	**C$_m$ELAAAMK**	**24-31**	**893.42**
Lys1	TPGSR	87-91	517.27

Table S1: Expected peptide fragments after complete tryptic digestion of Lysozyme from *gallus gallus.* In bold are the peptides observed by Aerolysin and CytK nanopores.*Cysteine residues are acetylated using iodoacetamide forming carbamidomethyl-cysteine (+57.02 Da mass shift compared to unmodified cysteine).

Peptide	Name	Mass	Length	m/z	RT (min)	Area 2-Digest	Accession	PTM
NLC(+57.02)NIPC(+57.02)SALLSSDITASVNC(+57.02)AK	Lys10	2507.18	23	1254.60	60.7	8.95E+08	LYSC_CHICK	Carbamidomethylation
FESNFNTQATNR	Lys7	1427.64	12	714.83	28.3	7.86E+08	LYSC_CHICK	
GYSLGNWVC(+57.02)AAK	Lys6	1324.62	12	663.32	47.5	7.47E+08	LYSC_CHICK	Carbamidomethylation
NTDGSTDYGILQINSR	Lys9	1752.83	16	877.42	43.5	6.97E+08	LYSC_CHICK	
GTDVQAWIR	Lys5	1044.54	9	523.27	45.4	6.83E+08	LYSC_CHICK	
WWC(+57.02)NDGR	Lys4	992.39	7	497.20	28.7	3.57E+08	LYSC_CHICK	Carbamidomethylation
N(+.98)TDGSTDYGILQINSR	Lys9	1753.81	16	877.92	44.7	3.00E+08	LYSC_CHICK	Deamidation (NQ)
NTDGSTDYGILQIN(+.98)SR	Lys9	1753.81	16	877.92	44.7	2.32E+08	LYSC_CHICK	Deamidation (NQ)
SALLSSDITASVNC(+57.02)AK		1635.81	16	818.91	50.7	2.09E+08	LYSC_CHICK	Carbamidomethylation
NLC(+57.02)NIPC(+57.02)SALLSSDITASVN		2148.02	20	1075.02	70.2	1.75E+08	LYSC_CHICK	Carbamidomethylation
IVSDGDGM(+15.99)NAWVAWR	Lys8	1691.77	15	846.89	49.0	1.72E+08	LYSC_CHICK	Oxidation (M)
N(+.98)LC(+57.02)N(+.98)IPC(+57.02)SALLSSDITASVN(+.98)C(+57.02)AK	Lys10	2510.13	23	1256.10	62.0	1.31E+08	LYSC_CHICK	Deamidation (NQ); Carbamidomethylation
NLC(+57.02)N(+.98)IPC(+57.02)SALLSSDITASVNC(+57.02)AK	Lys10	2508.17	23	1255.10	64.2	1.17E+08	LYSC_CHICK	Carbamidomethylation; Deamidation (NQ)
IVSDGDGMNAWVAWR	Lys8	1675.78	15	838.90	56.8	1.15E+08	LYSC_CHICK	
FESNFN(+.98)TQATNR	Lys7	1428.63	12	715.32	26.3	8.82E+07	LYSC_CHICK	Deamidation (NQ)
NTDGSTDYGILQIN		1509.69	14	755.85	49.1	7.76E+07	LYSC_CHICK	
PPGMPYNR		930.44	8	466.22	10.6	6.84E+07		
GYSLGN(+.98)WVC(+57.02)AAK	Lys 6	1325.61	12	663.81	45.1	6.80E+07	LYSC_CHICK	Deamidation (NQ); Carbamidomethylation
SLGNWVC(+57.02)AAK		1104.54	10	553.28	38.0	4.85E+07	LYSC_CHICK	Carbamidomethylation
IVSDGNGMNAWVAWR		1674.79	15	838.41	55.7	4.12E+07	LYSC_CHICK	
WWC(+57.02)N(+.98)DGR	Lys4	993.38	7	497.70	24.8	3.97E+07	LYSC_CHICK	Carbamidomethylation; Deamidation (NQ)
C(+57.02)ELAAAMK	Lys 2	892.41	8	447.21	21.7	3.80E+07	LYSC_CHICK	Carbamidomethylation
GYSLGNWVC(+57.02)		1054.45	9	528.23	50.9	3.71E+07	LYSC_CHICK	Carbamidomethylation
NLC(+57.02)NIPC(+57.02)		889.38	7	445.70	30.7	3.55E+07	LYSC_CHICK	Carbamidomethylation
FESN(+.98)FNTQATNR		1428.63	12	715.33	25.8	3.35E+07	LYSC_CHICK	Deamidation (NQ)
IVSDGNGM(+15.99)NAWVAWR		1690.79	15	846.40	47.4	3.30E+07	LYSC_CHICK	Oxidation (M)
N(+.98)TDGSTDYGILQIN		1510.68	14	756.35	50.7	2.85E+07	LYSC_CHICK	Deamidation (NQ)
NLC(+57.02)NIPC(+57.02)SALLSSDITASVN(+.98)C(+57.02)AK	Lys10	2508.17	23	1255.10	63.5	2.69E+07	LYSC_CHICK	Carbamidomethylation; Deamidation (NQ)
N(+.98)LC(+57.02)N(+.98)IPC(+57.02)SALLSSDITASVN		2149.99	20	1076.01	65.7	2.40E+07	LYSC_CHICK	Deamidation (NQ); Carbamidomethylation

Table S2: Detected peptides in the mass-spectrometry measurement of trypsinated lysozyme. The columns indicate (from left to right): the sequence, the name (according to table S1), the mass (in Da), the length of the peptide, the retention time (RT) on the HPLC column, the peak area (Area 2-Digest), which relates to the concentration in the sample, the Accession code (LYSC_CHICK = lysozyme from *gallus gallus*), and any detected PTM in the peptide. Only the 30 peptides with the highest peak area are shown. Peptide lys7 is highlighted in green and deaminated forms of lys7 are highlighted in orange.

	μ_{lys4} (I_{ex}%)	σ_{lys4} (I_{ex}%)	μ_{lys5} (I_{ex}%)	σ_{lys5} (I_{ex}%)	Resolution
Aer^{K238D}	31.4 ± 0.4	1.67 ± 0.03	n.d.	n.d.	n.d.
$Aer^{K238D+A260F}$	54.4 ± 0.8	1.05 ± 0.18	58.5 ± 0.6	1.71 ± 0.31	2.98 ± 0.21
$Aer^{K238D+S264F}$	54.6 ± 1.0	0.94 ± 0.07	59.2 ± 1.2	0.62 ± 0.29	5.97 ± 1.28
$Aer^{K238D+Q268F}$	34.0 ± 0.3	0.87 ± 0.10	37.0 ± 0.4	2.32 ± 0.23	1.86 ± 0.13
$Aer^{K238D+S272F}$	41.2 ± 0.1	2.74 ± 0.21	48.7 ± 0.8	5.22 ± 0.28	1.87 ± 0.28

Table S3: Resolution of peptides in the phenylalanine mutants. μ_i indicates the mean and σ_i the standard deviation of the peptide blockades. Errors indicate the standard error between measurements in three different nanopores.

		Reversal potential	P(K)/P(Cl)	Net charge of the barrel
Aer^{Wt}	pH 7.5	-3.9 ± 1.4 mV	0.76 ± 0.07	0
Aer^{Wt}	pH 3.8	-12.9 ± 1.0 mV	0.38 ± 0.03	+7
Aer^{K238D}	pH 3.8	-8.8 ± 0.9 mV	0.53 ± 0.04	+2
Aer^{K238D}	pH 3.0	-11.4 ± 0.6 mV	0.43 ± 0.02	+10
$Aer^{K238D+A260F}$	pH 3.0	-9.3 ± 0.3 mV	0.50 ± 0.01	+10
$CytK^{Wt}$	pH 7.5	+0.4 ± 0.3 mV	1.02 ± 0.02	0
$CytK^{Wt}$	pH 3.8	-7.7 ± 0.9 mV	0.58 ± 0.03	+5
$CytK^{K128D}$	pH 3.8	+1.3 ± 0.7 mV	1.10 ± 0.05	+1
$CytK^{K128D}$	pH 3.0	-0.1 ± 0.7 mV	0.99 ± 0.05	+7
$CytK^{S126F+K128D}$	pH 3.0	+0.0 ± 0.1 mV	1.00 ± 0.02	+7

Table S4: Reversal potential measurements of aerolysin and CytK nanopores. The reversal potential was measured under asymmetric salt conditions with 0.5 M KCl in the *trans* compartment and 2M KCl in the *cis* compartment, buffered to the appropriated pH using Tris (pH 7.5) or citric acid and bis-tris propane (pH 3.0 and 3.8). The error represents the standard deviation between three measurements in different pores. The last column shows the calculated charge in the barrel at the given pH in 150 mM NaCl, as described in the Methods.

Table S5: Ionic current versus the voltage of aerolysin mutants in 1M KCl at pH 3.8

Voltage (mV)	Aer^{Wt} (pA)	Aer^{K238Q} (pA)	Aer^{K238D} (pA)	Aer^{K238W} (pA)	Aer^{K238F} (pA)
-100	-61.7 ± 4.2	-63.1 ± 1.3	-60.7 ± 0.6	-59.2 ± 1.1	-60.3 ± 6.7
-95	-57.8 ± 4.7	-59.4 ± 1.3	-57.9 ± 0.6	-56.0 ± 1.2	-57.4 ± 6.2
-90	-55.1 ± 3.5	-56.6 ± 1.4	-55.1 ± 0.7	-53.0 ± 1.2	-54.3 ± 5.6
-85	-52.0 ± 3.3	-53.5 ± 1.3	-52.2 ± 0.7	-50.0 ± 1.1	-51.2 ± 5.2
-80	-48.9 ± 3.0	-50.5 ± 1.3	-49.3 ± 0.7	-46.6 ± 0.5	-48.2 ± 4.8
-75	-45.8 ± 2.8	-47.4 ± 1.2	-46.4 ± 0.6	-43.7 ± 0.4	-45.2 ± 4.5
-70	-42.7 ± 2.5	-44.2 ± 1.0	-43.4 ± 0.5	-40.7 ± 0.4	-42.2 ± 4.1
-65	-39.6 ± 2.2	-41.3 ± 1.1	-40.5 ± 0.6	-38.0 ± 0.8	-39.2 ± 3.6
-60	-36.5 ± 2.0	-38.3 ± 1.1	-37.5 ± 0.6	-35.1 ± 0.7	-36.2 ± 3.3
-55	-33.4 ± 1.7	-35.2 ± 1.0	-34.5 ± 0.6	-32.0 ± 0.3	-33.3 ± 3.0
-50	-30.5 ± 1.6	-32.1 ± 1.0	-31.5 ± 0.5	-29.0 ± 0.2	-30.2 ± 2.5
-45	-27.4 ± 1.4	-29.0 ± 0.9	-28.5 ± 0.5	-26.2 ± 0.4	-27.3 ± 2.2
-40	-24.3 ± 1.1	-25.9 ± 0.8	-25.4 ± 0.5	-23.1 ± 0.1	-24.2 ± 1.8
-35	-21.3 ± 1.0	-22.7 ± 0.7	-22.2 ± 0.5	-20.2 ± 0.1	-21.2 ± 1.6
-30	-18.2 ± 0.9	-19.5 ± 0.7	-19.2 ± 0.4	-17.4 ± 0.3	-18.2 ± 1.3
-25	-15.2 ± 0.7	-16.4 ± 0.6	-16.1 ± 0.4	-14.3 ± 0.2	-15.2 ± 1.0
-20	-12.1 ± 0.5	-13.1 ± -.6	-12.9 ± 0.3	-11.4 ± 0.2	-12.1 ± 0.8
-15	-9.1 ± 0.4	-9.8 ± 0.5	-9.8 ± 0.3	-8.5 ± 0.3	-9.1 ± 0.5
-10	-6.1 ± 0.2	-6.6 ± 0.5	-6.6 ± 0.3	-5.7 ± 0.3	-6.1 ± 0.4
-5	-3.1 ± 0.1	-3.3 ± 0.4	-3.5 ± 0.2	-2.8 ± 0.3	-3.1 ± 0.2
0	-0.1 ± 0.5	-0.0 ± 0.4	-0.3 ± 0.2	0.1 ± 0.3	0.0 ± 0.1
5	3.0 ± 0.1	3.2 ± 0.4	2.9 ± 0.1	2.9 ± 0.3	2.9 ± 0.2
10	5.9 ± 0.2	6.5 ± 0.4	6.0 ± 0.1	5.7 ± 0.4	5.9 ± 0.3
15	9.0 ± 0.4	9.8 ± 0.4	9.2 ± 0.2	8.4 ± 0.4	8.8 ± 0.5
20	11.9 ± 0.5	13.0 ± 0.4	12.3 ± 0.1	11.3 ± 0.4	11.7 ± 0.6
25	14.8 ± 0.5	16.2 ± 0.5	15.4 ± 0.1	13.9 ± 0.3	14.6 ± 0.8
30	17.6 ± 0.7	19.3 ± 0.5	18.5 ± 0.1	16.6 ± 0.3	17.4 ± 0.9
35	20.5 ± 0.7	22.4 ± 0.5	21.5 ± 0.1	19.3 ± 0.4	20.1 ± 1.1
40	23.3 ± 0.8	25.5 ± 0.5	24.6 ± 0.1	22.0 ± 0.4	22.9 ± 1.2
45	25.9 ± 0.8	28.6 ± 0.6	27.6 ± 0.1	24.5 ± 0.3	25.5 ± 1.3
50	28.6 ± 0.8	31.6 ± 0.6	30.6 ± 0.1	27.1 ± 0.4	28.1 ± 1.5
55	31.2 ± 0.9	34.5 ± 0.7	33.6 ± 0.1	29.5 ± 0.3	30.7 ± 1.6
60	33.9 ± 1.1	37.4 ± 0.7	36.6 ± 0.2	31.9 ± 0.3	33.2 ± 1.7
65	36.7 ± 1.3	40.2 ± 0.7	39.5 ± 0.2	34.4 ± 0.2	35.7 ± 2.0
70	39.2 ± 1.2	43.0 ± 0.8	42.4 ± 0.3	36.8 ± 0.3	38.2 ± 2.0
75	41.6 ± 1.2	45.8 ± 0.8	45.2 ± 0.5	39.2 ± 0.4	40.5 ± 2.2
80	44.2 ± 1.3	48.4 ± 0.9	48.2 ± 0.4	41.5 ± 0.3	42.9 ± 2.3
85	46.7 ± 1.5	51.1 ± 1.0	51.1 ± 0.5	43.8 ± 0.3	45.2 ± 2.5
90	49.1 ± 1.5	53.8 ± 1.1	54.0 ± 0.6	46.1 ± 0.3	47.6 ± 2.7
95	51.6 ± 1.8	56.3 ± 1.2	57.0 ± 0.6	48.3 ± 0.4	49.9 ± 2.8
100	54.3 ± 2.0	59.2 ± 1.3	60.4 ± 0.6	50.9 ± 0.5	52.6 ± 3.1

Table S6: Ionic current versus the voltage of CytK mutants in 1M KCl at pH 3.8

Voltage (mV)	$CytK^{Wt}$ (pA)	$CytK^{K128F}$ (pA)	$CytK^{K128D}$ (pA)	$CytK^{S126F}$ (pA)
-100	-110.1 ± 1.8	-98.0 ± 1.1	-99.9 ± 1.4	-112.8 ± 0.5
-95	-104.4 ± 1.7	-93.6 ± 0.9	-95.5 ± 1.4	-106.5 ± 0.4
-90	-98.5 ± 1.7	-89.2 ± 0.9	-91.5 ± 1.3	-100.0 ± 0.6
-85	-92.6 ± 1.7	-84.7 ± 1.0	-86.9 ± 1.4	-94.3 ± 0.4
-80	-87.0 ± 1.6	-80.2 ± 1.0	-82.4 ± 1.3	-87.4 ± 1.7
-75	-81.3 ± 1.5	-75.6 ± 0.8	-77.9 ± 1.3	-82.3 ± 0.3
-70	-75.6 ± 1.5	-71.0 ± 0.8	-73.3 ± 1.2	-76.4 ± 0.3
-65	-69.9 ± 1.5	-66.3 ± 0.9	-68.6 ± 1.1	-70.5 ± 0.3
-60	-64.4 ± 1.4	-61.6 ± 0.8	-63.8 ± 1.1	-64.8 ± 0.3
-55	-58.8 ± 1.4	-56.8 ± 0.8	-59.0 ± 0.9	-59.1 ± 0.3
-50	-53.3 ± 1.3	-52.0 ± 0.8	-54.0 ± 0.9	-53.4 ± 0.2
-45	-47.8 ± 1.3	-47.2 ± 0.7	-49.0 ± 0.9	-47.8 ± 0.2
-40	-42.4 ± 1.2	-42.1 ± 0.7	-43.9 ± 0.8	-42.3 ± 0.2
-35	-37.1 ± 1.2	-37.3 ± 0.7	-38.7 ± 0.8	-36.9 ± 0.2
-30	-31.7 ± 1.1	-32.3 ± 0.7	-33.4 ± 0.7	-31.5 ± 0.2
-25	-26.4 ± 1.0	-27.3 ± 0.6	-28.0 ± 0.7	-26.1 ± 0.2
-20	-21.1 ± 1.0	-22.2 ± 0.6	-22.6 ± 0.6	-20.9 ± 0.2
-15	-15.9 ± 0.9	-17.1 ± 0.6	-17.1 ± 0.6	-15.7 ± 0.1
-10	-10.7 ± 0.9	-12.0 ± 0.5	-11.5 ± 0.5	-10.6 ± 0.2
-5	-5.5 ± 0.8	-6.7 ± 0.5	-5.9 ± 0.5	-5.5 ± 0.2
0	-0.4 ± 0.8	-1.5 ± 0.5	-0.2 ± 0.4	-0.4 ± 0.2
5	4.7 ± 0.7	3.8 ± 0.5	5.6 ± 0.4	4.6 ± 0.2
10	9.7 ± 0.6	9.1 ± 0.5	11.5 ± 0.3	9.5 ± 0.2
15	14.7 ± 0.6	14.4 ± 0.5	17.4 ± 0.3	14.4 ± 0.2
20	19.6 ± 0.5	19.8 ± 0.4	23.3 ± 0.3	19.2 ± 0.2
25	24.5 ± 0.5	25.2 ± 0.4	29.4 ± 0.3	24.0 ± 0.2
30	29.3 ± 0.4	30.7 ± 0.4	35.4 ± 0.3	28.8 ± 0.3
35	34.1 ± 0.4	36.1 ± 0.4	41.6 ± 0.3	33.4 ± 0.3
40	38.8 ± 0.4	41.6 ± 0.4	47.8 ± 0.3	37.9 ± 0.3
45	43.5 ± 0.4	47.1 ± 0.2	54.0 ± 0.3	42.4 ± 0.4
50	48.0 ± 0.4	52.6 ± 0.1	60.3 ± 0.3	46.9 ± 0.4
55	52.8 ± 0.5	58.1 ± 0.1	66.6 ± 0.4	51.3 ± 0.5
60	57.2 ± 0.5	63.8 ± 0.2	73.0 ± 0.4	55.7 ± 0.5
65	61.8 ± 0.6	69.4 ± 0.2	79.5 ± 0.5	59.9 ± 0.6
70	66.2 ± 0.7	75.1 ± 0.3	86.0 ± 0.5	64.2 ± 0.6
75	70.7 ± 0.8	80.0 ± 1.4	92.5 ± 0.5	68.3 ± 0.7
80	75.2 ± 1.3	86.5 ± 0.4	99.1 ± 0.6	71.9 ± 1.0
85	79.4 ± 1.0	92.3 ± 0.4	105.7 ± 0.7	76.6 ± 0.9
90	83.8 ± 1.0	98.2 ± 0.5	112.4 ± 0.8	80.6 ± 1.0
95	88.1 ± 0.9	104.2 ± 0.5	119.3 ± 0.9	84.7 ± 1.3
100	92.8 ± 0.9	111.1 ± 0.4	126.8 ± 1.0	88.9 ± 1.9

Amino acid sequence of pro-Aer^{Wt}

MAEPVYPDQLRLFSLGQGVCGDKYRPVNREEAQSVKSNIVGMMGQWQISGLANGWVIMG
PGYNGEIKPGTASNTWCYPTNPVTGEIPTLSALDIPDGDEVDVQWRLVHDSANFIKPTSYLA
HYLGYAWVGGNHSQYVGEDMDVTRDGDGWVIRGNNDGGCDGYRCGDKTAIKVSNFAYN
LDPDSFKHGDVTQSDRQLVKTVVGWAVNDSDTPQSGYDVTLRYDTATNWSKTNTYGLSEK
VTTKNKFKWPLVGETELSIEIAANQSWASQNGGSTTTSLSQSVRPTVPARSKIPVKIELYKAD
ISYPYEFKADVSYDLTLSGFLRWGGNAWYTHPDNRPNWNHTFVIGPYKDKASSIRYQWDK
RYIPGEVKWWDWNWTIQQNGLSTMQNNLARVLRPVRAGITGDFSAESQFAGNIEIGAPVPL
AADSKVRRARSVDGAGQGLRLEIPLDAQELSGLGFNNVSLSVTPAANQGSSHHHHHH

DNA sequence of pro-Aerolysin gene

ATGGCGGAGCCGGTCTATCCGGATCAACTCCGGCTCTTCTCATTAGGCCAGGGTGTCT
GCGGTGACAAATATCGTCCTGTTAATCGGGAGGAGGCTCAATCGGTCAAATCAAACATC
GTGGGTATGATGGGCCAATGGCAGATTAGTGGCCTGGCCAACGGTTGGGTAATTATGG
GGCCGGGGTACAACGGCGAGATCAAACCTGGCACCGCGTCGAACACTTGGTGCTACC
CTACTAATCCAGTGACAGGTGAGATTCCAACCTTGTCAGCGCTCGATATCCCGGACGGC
GACGAAGTGGATGTTCAGTGGCGCCTTGTTCATGACAGTGCGAATTTCATCAAACCTAC
TAGTTACTTAGCCCATTATTTGGGGTATGCGTGGGTTGGCGGCAACCACAGTCAGTAT
GTAGGGGAAGACATGGATGTTACACGGGACGGCGACGGCTGGGTGATCCGCGGGAAC
AACGACGGGGGCTGTGACGGCTACCGTTGCGGCGACAAAACGGCGATTAAGGTGTCC
AATTTTGCATATAACCTTGATCCTGATTCGTTCAAACACGGGGACGTGACGCAAAGTGA
CCGCCAACTCGTAAAGACTGTTGTAGGCTGGGCTGTGAACGACAGTGACACGCCACAG
AGTGGCTACGACGTTACTCTTCGTTACGACACGGCAACGAACTGGTCTAAAACGAACAC
TTACGGTCTCTCTGAAAAAGTCACTACCAAAAACAAGTTCAAGTGGCCGTTGGTAGGCG
AAACAGAACTCAGCATCGAAATCGCAGCCAACCAAAGTTGGGCTAGTCAAAACGGCGG
GAGCACAACGACGTCGCTGAGTCAATCCGTCCGCCCAACTGTGCCTGCTCGTTCCAAG
ATCCCAGTCAAAATTGAATTATATAAAGCTGATATTTCTTACCCTTATGAATTTAAGGCAG
ATGTTAGTTACGACCTGACGTTATCGGGGTTCCTCCGCTGGGGTGGTAATGCTTGGTAT
ACTCACCCGGATAACCGTCCAAACTGGAATCACACGTTCGTGATCGGTCCGTACAAAGA
TAAGGCGAGCTCTATCCGCTATCAATGGGACAAGCGCTATATCCCTGGCGAAGTTAAAT
GGTGGGACTGGAATTGGACTATCCAACAGAACGGCCTCTCTACAATGCAAAACAACCTG
GCCCGCGTACTGCGGCCAGTTCGGGCGGGCATTACGGGGGACTTCTCCGCAGAAAGC
CAGTTTGCCGGCAATATCGAAATCGGGGCACCGGTTCCTCTCGCCGCTGATTCTAAGGT
ACGTCGGGCACGGTCCGTGGACGGTGCGGGGCAGGGGTTGCGTCTGGAGATTCCGCT
TGACGCCCAAGAGTTGTCGGGGTTAGGTTTTAACAATGTGTCCTTGAGTGTCACACCAG
CCGCTAACCAAGGGTCCTCTCATCACCATCACCACCAC

DNA sequence of CytK gene

CAAAAACCATGGCGCAAACGACTTCACAGGTTGTAACAGATATAGGCCAGAATGCTAAA
ACCCATACCAGCTACAATACCTTCAACAACGAACAAGCGGACAACATGACCATGAGCCT
GAAGGTGACGTTCATCGATGATCCGAGCGCAGACAAACAAATTGCGGTTATCAACACCA
CCGGCTCGTTCATGAAAGCAAATCCGACTCTGAGCGACGCACCGGTTGATGGTTACCC
GATTCCGGGCGCGTCTGTGACCCTGCGCTATCCGAGCCAATATGATATTGCCATGAACC
TCCAGGACAATACCTCGCGCTTTTTCCACGTGGCGCCAACGAACGCTGTCGAGGAAAC
GACTGTTACCAGCAGTGTCTCCTACCAGTTGGGTGGTTCTATCAAGGCTAGCGTGACCC
CGTCCGGCCCTTCTGGTGAGTCCGGTGCGACCGGTCAGGTTACCTGGTCAGATTCCGT
TAGCTACAAACAAACCTCCTACAAGACGAACCTGATCGACCAGACGAACAAGCACGTG
AAATGGAACGTGTTTTTTAACGGCTACAACAATCAAAACTGGGGTATTTATACCCGTGAT
AGCTACCATGCGCTGTATGGCAATCAGTTGTTTATGTATAGCCGTACCTATCCGCATGA
GACGGATGCGCGTGGTAACCTTGTTCCAATGAACGATCTGCCGACTTTGACCAACAGC
GGCTTCAGCCCGGGTATGATCGCGGTTGTTATTAGCGAAAAGGACACCGAACAGAGCT
CGATCCAGGTAGCCTATACCAAACACGCTGACGACTACACCTTACGTCCGGGCTTTACC
TTTGGTACAGGCAACTGGGTTGGTAATAACATCAAGGACGTGGACCAAAAAACTTTCAA
TAAGTCTTTCGTGCTGGATTGGAAAAATAAGAAGCTGGTCGAGAAAAAGGGCTCCGCGC
ATCAGCAGCATCATCAGTAG

Amino acid sequence of CytK^{Wt}

MAQTTSQVVTDIGQNAKTHTSYNTFNNEQADNMTMSLKVTFIDDPSADKQIAVINTTGSFMK
ANPTLSDAPVDGYPIPGASVTLRYPSQYDIAMNLQDNTSRFFHVAPTNAVEETTVTSSVSYQ
LGGSIDASVTPSGPSGESGATGQVTWSDSVSYKQTSYKTNLIDQTNKHVKWNVFFNGYNN
QNWGIYTRDSYHALYGNQLFMYSRTYPHETDARGNLVPMNDLPTLTNSGFSPGMIAVVISE
KDTEQSSIQVAYTKHADDYTLRPGFTFGTGNWVGNNIKDVDQKTFNKSFVLDWKNKKLVEK
KGSAHHHHHH
